# Photocatalytic and Photo-Fenton Degradation Activity of Hierarchically Structured α-Fe_2_O_3_@Fe-CeO_2_ and g-C_3_N_4_ Composite

**DOI:** 10.3390/ijms27073133

**Published:** 2026-03-30

**Authors:** Aneta Bužková, Radka Pocklanová, Vlastimil Novák, Martin Petr, Barbora Štefková, Alexandra Rancová, Josef Kašlík, Robert Prucek, Aleš Panáček, Libor Kvítek

**Affiliations:** 1 Department of Physical Chemistry, Faculty of Science, Palacký University, 17. listopadu 1192/12, 779 00 Olomouc, Czech Republic; aneta.buzkova@upol.cz (A.B.); barbora.stefkova@upol.cz (B.Š.); robert.prucek@upol.cz (R.P.); ales.panacek@upol.cz (A.P.); 2 Department of Chemistry and Physico-Chemical Processes, Faculty of Materials Science and Technology, Technical University of Ostrava, 17. listopadu 2172/15, 708 00 Ostrava-Poruba, Czech Republic; 3 Regional Centre of Advanced Technologies and Materials (RCPTM), Czech Advanced Technology and Research Institute (CATRIN), Palacký University, Šlechtitelů 241/27, 779 00 Olomouc, Czech Republic

**Keywords:** photocatalysis, Photo-Fenton, g-C_3_N_4_, iron doped cerium oxide, hematite, degradation, Rhodamine B

## Abstract

The hematite phase decorated with iron-doped cerium oxide nanoparticles (F@FC) was precipitated from cerium and iron oxalate intermediate products. The photocatalytic composite of graphitic carbon nitride (gCN) and F@FC was prepared by a simple method involving mixing the two components, followed by thermal treatment at 400 °C. According to electron microscopy, F@FC is composed of a submicron iron oxide (hematite) phase decorated with iron-doped cerium oxide nanoparticles deposited on gCN substrate. A hierarchically structured composite was observed instead of a simple mechanical mixture of α-Fe_2_O_3_, Fe-CeO_2_, and gCN. To observe two types of degradation activity, photocatalytic and Photo-Fenton degradation activity, Rhodamine B (RhB) was applied as the model water pollutant. The influence of the amount of photocatalyst, the RhB concentration, the presence of cations and anions, the pH, and the effect of e^−^, h^+^, •OH, and •O_2_^−^ scavenging reactants were studied. The Photo-Fenton degradation exhibited high efficiency across the entire tested pH range, whereas photocatalytic degradation showed comparable activity only at acidic pH. The F@FC-gCN composite catalyst exhibited a high degree of recyclability. The degradation pathways of photocatalytic and Photo-Fenton reactions were suggested by HPLC-MS analysis of the reaction products. A notable finding of this study was the observation that the green-yellow, fluorescent intermediate Rhodamine 110 was formed during the photocatalytic degradation of RhB. However, the high reactivity of the generated •OH radicals during Photo-Fenton degradation has been demonstrated to inhibit the formation of intermediate Rhodamine 110.

## 1. Introduction

Water contamination by heavy metals [1], pesticides [2], pharmaceuticals [3], microplastics [4], and pathogens [5] is a critical environmental issue that demands the study of novel, innovative water treatment materials and processes in addition to conventional methods, such as sedimentation, coagulation, flocculation, and filtration [6]. Advanced oxidation processes (AOPs) are a class of innovative water treatment technologies developed to effectively remove organic pollutants and pathogens from water sources. These processes utilize highly reactive oxygen species (ROS), e.g., hydroxyl (•OH) and superoxide (•O_2_^−^) radicals, to remove contaminants that are often resistant to conventional water treatment methods [7]. Fenton, Photo-Fenton, and photocatalytic degradation are typical examples of AOPs that achieve excellent results in the elimination of organic pollutants [8,9]. Photodegradation is a process that uses UV-Vis light for the formation of the electron–hole pair in a photocatalytic material. These charge carriers can generate ROS from water at the photocatalyst surface, enabling the degradation of pollutant molecules [10,11]. Fenton and Photo-Fenton degradation use Fenton’s reagent, which is a mixture of oxidizing agent (e.g., hydrogen peroxide [12,13], peroxymonosulfate [14,15]) and iron species [16,17,18]. In Fenton degradation, iron (Fe^2+^) reacts with hydrogen peroxide to produce hydroxyl radicals and ferric ions (Fe^3+^), as stated:

(1) $${Fe}^{2+}+H_{2}O_{2}\to {Fe}^{3+}+•OH+{OH}^{-}$$

The hydroxyl radicals (•OH) are highly reactive and can oxidize various organic pollutants. Both homogeneous and heterogeneous Photo-Fenton degradation processes additionally use UV-Vis light to reduce Fe^3+^ back to Fe^2+^ through the following reactions:

(2) $${Fe}^{3+}+H_{2}O+hv\to {Fe}^{2+}+•OH+H^{+}$$

(3) $${Fe}^{3+}+H_{2}O_{2}+hv\to {Fe}^{2+}+•OOH+H^{+}$$

One of the key advantages of Photo-Fenton degradation is the cyclic oxidation and reduction of iron, which ensures continuous production of active species during the treatment process [16,17,18]. Moreover, unlike conventional methods, AOPs can completely mineralize pollutants after prolonged light exposure, producing harmless byproducts such as carbon dioxide and water [9]. However, it has been demonstrated that intermediate products with higher toxicity than the original pollutant molecules can be formed during the degradation process [19,20,21,22,23].

The photodegradation activity of photocatalysts can be influenced by various factors, including the intensity and spectrum of the light, the pH of the system, and the presence of other molecules. The most studied photocatalysts in water treatment technologies are TiO_2_, ZnO [24], BiVO_4_ [25], and gCN (graphitic carbon nitride) [26]. Pure gCN can absorb light up to 450 nm [26]. However, with appropriate modification, it is possible to achieve gCN absorption at even longer wavelengths and to improve other photocatalytic properties, such as electron–hole pair recombination [26]. A common modification of gCN is doping with metals and nonmetals, vacancy creation [27], or forming composite materials with metals and their oxides, sulfides, and ferrites [26]. Cerium oxide is widely used in oxidation catalysis [28]. However, the high band gap energy of 3.0–3.2 eV in pure CeO_2_ limits its photocatalytic activity, as it is incapable of effectively utilizing visible light. To enhance visible light absorption and overall photocatalytic activity, defect engineering through doping is commonly employed to narrow the band gap [29,30,31]. Furthermore, the construction of heterojunctions (e.g., with hematite) has been shown to be an effective strategy for enhancing the charge-separation efficiency of cerium oxide [32,33].

This study focuses on the preparation and effectiveness evaluation of a composite containing hematite decorated with iron-doped cerium oxide and graphitic carbon nitride for photocatalytic and Photo-Fenton degradation processes. The synthesis of α-Fe_2_O_3_@Fe-CeO_2_ was accomplished by precipitating cerium oxalate and iron oxalate in the presence of a complexing agent, tetraethylammonium hydroxide. The final composite F@FC-gCN was prepared by simple mixing of the primary prepared F@FC and gCN, followed by thermal treatment. The photocatalytic and Photo-Fenton degradation activities were studied through Rhodamine B degradation under white light irradiation. Moreover, the potential degradation pathway of RhB in the studied reactions was proposed using HPLC-MS analysis.

## 2. Results and Discussion

### 2.1. Chemical Structure

**Figure 1:**
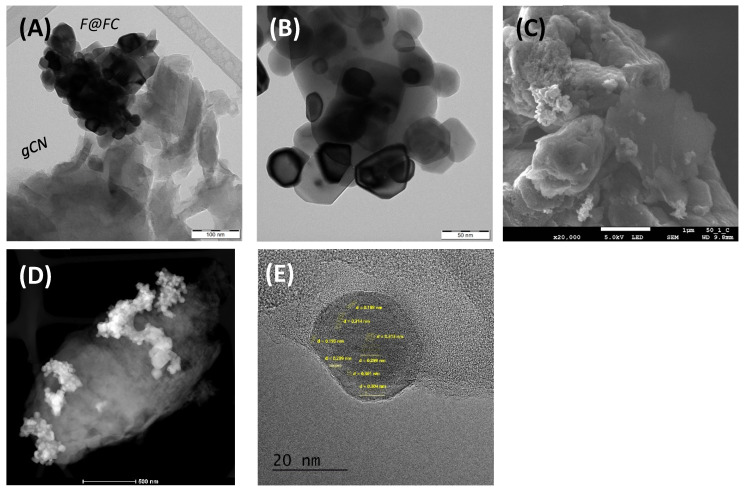
(**A**) TEM of the F@FC-gCN (1:15) composite and (**B**) a closer view of the CeO_2_ nanoparticles in the F@FC material. (**C**) SEM and (**D**) STEM image of the F@FC-gCN composite. (**E**) HRTEM image of the isolated CeO_2_ nanoparticle surrounded by gCN layers.

**Figure 2:**
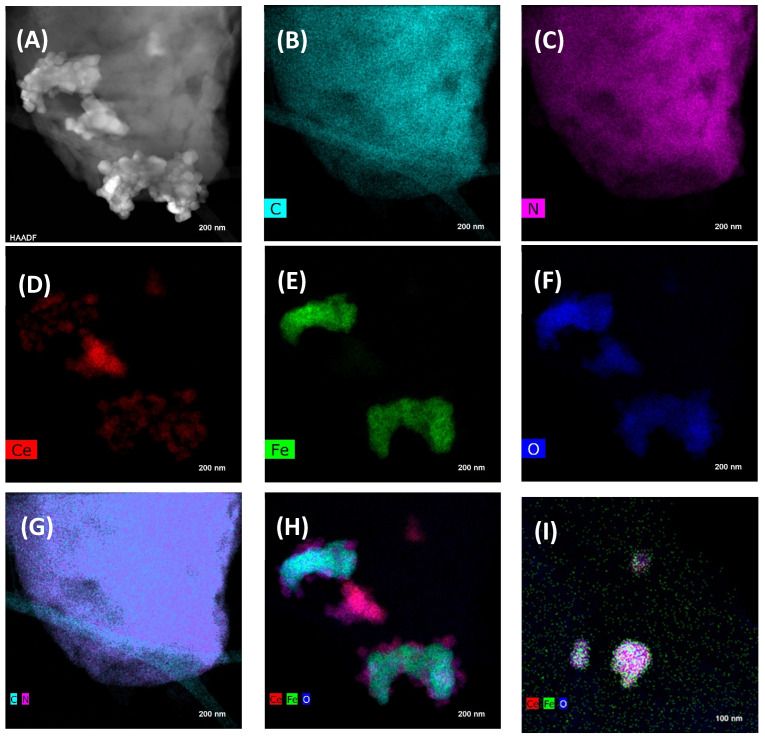
(**A**) HAADF image and elemental mapping of the F@FC-gCN composite of (**B**) carbon, (**C**) nitrogen, (**D**) cerium, (**E**) iron, (**F**) oxygen, (**G**) carbon and nitrogen, (**H**) cerium, iron, and oxygen. (**I**) Elemental mapping of cerium, iron, and oxygen of the CeO_2_ nanoparticles doped with iron in the F@FC-gCN composite; for more, see Figure S3.

**Figure 3:**
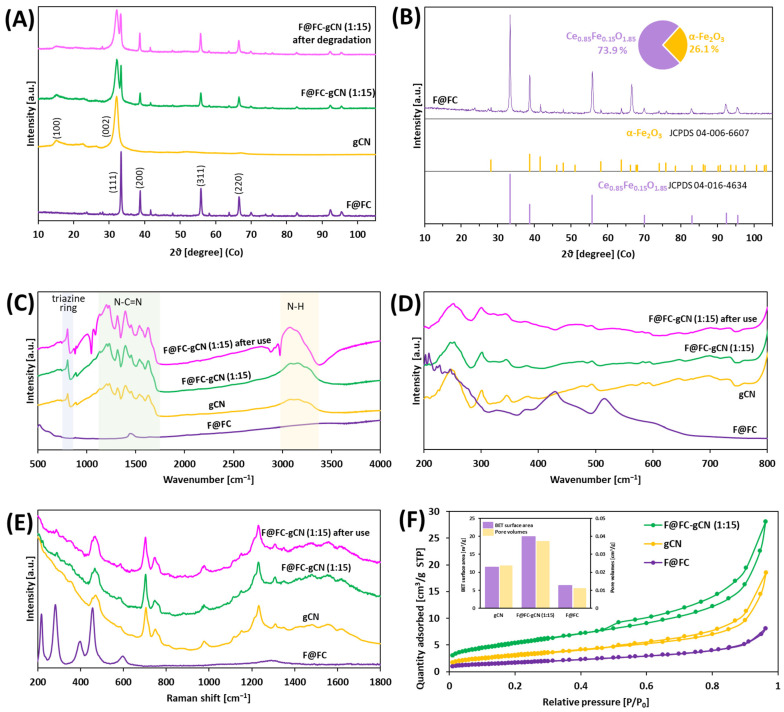
(**A**) X-ray diffraction patterns and (**B**) enlarged XRD spectrum of pure F@FC phase with corresponding JCPDS cards and Rietveld analysis. ATR-IR spectra using an IR source in a (**C**) MID region and a (**D**) FAR region. (**E**) Raman spectra. (**F**) N_2_ adsorption–desorption isotherms with pore volumes and BET specific surface area.

The morphology of the hematite decorated with iron-doped cerium oxide nanoparticles (F@FC) and its hierarchical composite with graphitic carbon nitride (gCN) was characterized using several methods. The structural characteristics of the prepared samples were examined through transmission electron microscopy (TEM) and scanning electron microscopy (SEM) (see Figure 1 and Figure S1). The TEM and SEM images illustrate the layered bulk structure of pure gCN (see Figure S1A,E,F). The energy-dispersive spectroscopy (EDS) analysis (Figure S2A) confirmed that gCN exclusively consists of carbon and nitrogen atoms. The TEM and SEM revealed the presence of hematite phase decorated with CeO_2_ nanoparticles in the pure F@FC sample (Figure S1B,G). The size of the CeO_2_ nanoparticles ranges from 20 to 125 nm (see histogram at Figure S1I,J), with the majority of particles measuring between 23 and 43 nm. The EDS analysis of F@FC (Figure S2C) confirmed the presence of iron, oxygen, and cerium atoms, along with residual carbon atoms. These carbon atoms likely originated from oxalic acid and TEAH employed during the synthetic process. High-resolution transmission electron microscopy (HRTEM) analysis confirms the high crystallinity of the CeO_2_ nanoparticle embedded within the graphitic carbon nitride matrix, as evidenced by the well-defined lattice fringes (Figure 1E). The observed lattice fringes exhibit *d* spacing values in the range of 0.299 to 0.314 nm and 0.189 to 0.193 nm, which can be indexed to the (111) and (220) crystal planes of the cubic CeO_2_ structure, respectively. The slight contraction observed in some (111) *d*-spacings is consistent with the lattice strain induced by the substitution of Ce^4+^ ions with smaller Fe^3+^ cations due to the iron doping within the CeO_2_ lattice. These observations are consistent with the XRD patterns discussed below. In the composite material, F@FC is conjugated with a gCN structure (Figure 1). As indicated by the high-angle annular dark field (HAADF) elemental mapping (Figure 2), the F@FC cluster consists of hematite phase decorated with cerium oxide nanoparticles. However, further investigation of isolated CeO_2_ nanoparticles on the gCN surface revealed the presence of iron doping within CeO_2_ nanoparticles (Figure 2I or Figure S3). Those isolated CeO_2_ nanoparticles probably occurred as a result of mixing during composite preparation. It is noteworthy that the presence of iron doping within CeO_2_ nanoparticles was not distinguishable during the initial mapping (Figure 2A–H). This phenomenon is likely attributable to detector saturation by the robust signal originating from the adjacent hematite. Prolonged exposure would probably resolve the iron doping. Hence, the synthesized F@FC-gCN composite can be characterized as a hierarchically structured material, as it exhibits a well-defined organization across multiple length scales. This hierarchical architecture comprises three distinct structural levels: nanoscopic Fe-doped CeO_2_ nanoparticles, mesoscopic hematite phase, and bulk layered gCN. The outcomes of electron microscopy align with those of X-ray diffraction (XRD) measurements. As illustrated in Figure 3A, within the pure F@FC sample, the predominant XRD pattern corresponds to the Ce_0.85_Fe_0.15_O_1.85_ phase (JCPDS Card No. 04-016-4634), exhibiting characteristic peaks at 2θ positions of 33.3, 38.7, 55.8, 70.0, 83.0, 92.5, and 95.5°. This phase corresponds to the cubic structure of cerium oxide, in which tetravalent Ce^4+^ cations in the CeO_2_ lattice are substituted by trivalent Fe^3+^ ions. To maintain overall electroneutrality in the formed crystal, oxygen vacancies are generated, leading to partial reduction in Ce^4+^ to Ce^3+^ [34,35]. This corresponds to the XPS analysis discussed later in this study. Moreover, this iron-doped cerium oxide phase constitutes 73.9 wt.% of the F@FC sample, as determined through quantitative phase analysis via Rietveld refinement (Figure 3B). The remaining 26.1 wt.% is attributed to the hematite α-Fe_2_O_3_ phase (JCPDS Card No. 04-006-6607). The mean crystallite size of iron-doped cerium oxide was determined to be 41 nm, which corresponds to the histograms obtained from the TEM and SEM analyses (Figure S1I,J). The XRD patterns obtained for the pure gCN (ICPDS Card No. 04-87-1526) exhibited two sharp peaks. The prominent peak occurring at 2θ 32.1° corresponds to the (0 0 2) crystal lattice, with an interplanar distance of aromatic units 0.168 nm. This peak is associated with the interlayer stacking of the conjugated aromatic layers. The smaller peak at 15.2° corresponds to the (1 0 0) crystal lattice, with an in-planar distance of *d* = 0.341 nm. The F@FC-gCN (1:15) composite exhibited the combined XRD patterns of pure gCN and pure F@FC. Following photodegradation, the used F@FC-gCN (1:15) composite was remeasured. No observable changes were observed within the XRD spectrum.

In the infrared (IR) spectra (Figure 3C), two typical vibration bands occurred for the pure gCN. The broad vibrational band within the 1100 cm^−1^ to 1600 cm^−1^ range is typical for aromatic CN heterocycles, which can be interpreted as the N-C=N stretching. The second broad vibrational band in the range from 3000 to 3400 cm^−1^ corresponds to the N-H stretching of primary and secondary amides. The sharp peak observed at 804 cm^−1^ is indicative of the vibration of the triazine ring. The absence of triple bond C≡N vibrations around 2200 cm^−1^ indicates the absence of any structure vacancies [36,37]. For the F@FC, a single peak at the vibration of 1440 cm^−1^ has been observed (Figure 3A and Figure S4A), which can be attributed to the C-H stretch. This is likely due to residues from oxalic acid and TEAH used during the synthetic process. This finding aligns with the observations made by EDS analysis. The F@FC IR spectrum, measured with a FAR IR source (see Figure 3D), exhibited two peaks at 430 cm^−1^ and 516 cm^−1^. The observed peaks are attributed to the metal–oxygen stretching modes, Ce-O and Fe-O, respectively [13,38]. However, these vibrations are not observed in the spectra of composite materials, likely due to the predominant gCN vibrations.

Subsequently, the prepared samples were subjected to Raman spectroscopy analysis (see Figure 3E). The pure gCN exhibits distinct peaks at 471, 706, 751, 985, and 1233 cm^−1^, which are characteristic vibrational modes of the graphitic carbon nitride lattice. This finding is consistent with the previous Raman studies of gCN [39]. Furthermore, variations in the relative intensities of the Raman bands at 706 and 751 cm^−1^ are considered indicators of morphological changes in gCN. According to the literature, a decrease in the intensity of these bands is associated with the transformation of bulk gCN into exfoliated nanosheet structures [39]. The Raman spectrum of pure F@FC exhibits multiple peaks. The cubic CeO_2_ structure exhibits a characteristic prominent peak at 456 cm^−1^ [40]. The iron-doped cerium oxide lattice generally exhibits minor 397 and 599 cm^−1^ peaks, attributable to oxygen vacancies within the CeO_2_ lattice [29,30]. The remaining prominent peaks at 216 and 281 cm^−1^, as well as the increased intensity at 397 cm^−1^ and the minor peak at 1291 cm^−1^, are attributed to hematite Raman modes [41]. In the F@FC-gCN composites, no significant decrease in the intensities of these bands was observed when compared to the pure gCN. However, the presence of peaks at 296 and 447 cm^−1^ was observed, indicating the incorporation of an F@FC component into the gCN phase. The intensity of these two bands declines with decreasing amount of F@FC in composites (Figure S4B). After photodegradation, the F@FC-gCN (1:15) composite was analyzed, revealing no changes in the Raman spectrum.

The surface area and pore volumes (Figure 3F) were determined using nitrogen gas sorption. The pure carbon nitride exhibited a surface area of 11.5 m^2^/g and a pore volume of 0.024 cm^3^/g. The specific surface area of F@FC was lower at 6.3 m^2^/g, with a pore volume of 0.011 cm^3^/g. The smaller surface area resulting from physical sorption is consistent with SEM images, which show the smooth surface of F@FC with low porosity. Meanwhile, an increase in surface area was observed for all four prepared composite materials (see Figure S4C). The F@FC-gCN (1:15) composite exhibited the highest surface area of 19.8 m^2^/g and a pore volume of 0.037 cm^3^/g. This increase most likely occurred due to mixing and homogenization of F@FC and gCN powders during the preparation of the F@FC-gCN composite. The isotherm of type IV and hysteresis loop H3 [42] (Figure 3F and Figure S4C), indicating a mesoporous structure, was observed for all samples. Compared to pure gCN and pure F@FC, the increased surface area of the composites could also contribute to the enhancement of photocatalytic activity, among other contributions. The ZPC was established by measuring the zeta potential of the dispersed material in water as a function of pH. The zero point charge (ZPC) of F@FC-gCN (1:15) was obtained at a pH of 4.21 (see Figure S4D).

**Figure 4:**
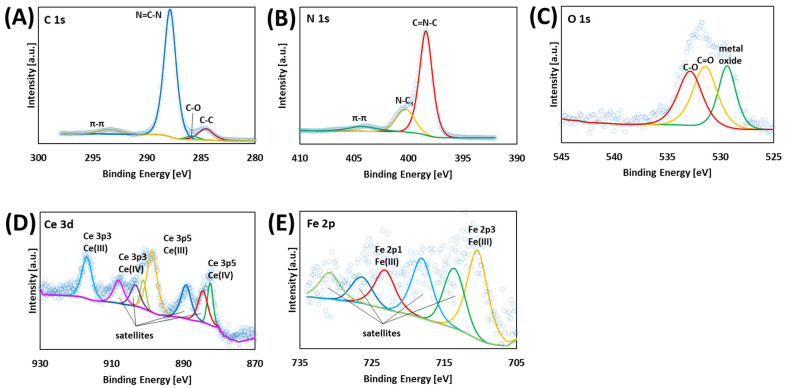
The deconvolution of XPS spectra for the F@FC-gCN (1:15) composite material of (**A**) C 1s, (**B**) N 1s, (**C**) O 1s, (**D**) Ce 3d, and (**E**) Fe 2p. Experimental data are represented by blue circles, while the overall fit and individual deconvoluted components are shown as solid-colored lines.

The chemical states of the elements were investigated by X-ray photoelectron spectroscopy (XPS). As illustrated in Figure S5C, the survey spectrum of the F@FC-gCN composite exhibits peaks at 881 eV (Ce 4s), 709 eV (Fe 2p3), 530 eV (O 1s), 400 eV (N 1s), and 288 eV (C 1s). Peak deconvolution of the Fe 2p3 for pure F@FC (Figure S6) and F@FC-gCN composite (Figure 4) reveals exclusively the 3+ oxidation state of the iron within iron-doped cerium oxide as well as the hematite phase. Moreover, cerium was found to exist in two distinct oxidation states: 3+ and 4+. Therefore, the partial reduction of Ce^4+^ to Ce^3+^ in the CeO_2_ lattice, resulting from oxygen vacancies caused by iron doping in the cerium oxide lattice, has been confirmed. The dual oxidation state of cerium opens up its redox potential. The O 1s spectra exhibited peaks at 531.44 eV and 529.37 eV, attributed to C=O and metal oxide, respectively. The C 1s spectrum of pure gCN (Figure S8) and F@FC-gCN composite (Figure 4) reveals a prominent peak at 287.88 eV, which is typical for sp^2^ hybridized carbon in triazine ring N=C-N. Additionally, peaks for C-C and π excitations were detected at 284.57 eV and 293.43 eV, respectively. A minor peak at 285.85 eV was attributed to C-O, which aligns with the O 1s spectra (Figure 4C) at a binding energy of 532.85 eV. However, since the intensity of the C-O structure is very low, the oxygen in pure gCN is likely due to exposure to air and humidity. The prominent peak at 398.39 eV in N 1s can be ascribed to sp^2^ hybridized nitrogen in the triazine ring C=N-C, while the peak at 400.3 eV corresponds to tertiary nitrogen N-C_3_. A minor peak at 404.2 eV was also observed for π excitations. The carbon-to-nitrogen ratio in pure gCN was determined to be 0.69, which is lower than the theoretical C/N ratio of 0.75 [43,44]. The shift in binding energies in the XPS spectrum (measured in dark) was evaluated to investigate the electron transfer and the interface of the F@FC-gCN (1:15) composite. The analysis revealed a clear redistribution of electron density at the heterojunction interface. A positive shift was observed in the constitutive elements of the F@FC phase, including Fe 2p3, Ce 4s, and O 1s, when comparing the pure F@FC to the composite. Specifically, the Ce 3p5 (III) peak shifted by +1.17 eV, the Ce 3p5 (IV) peak shifted by +0.77 eV, the Fe 2p3 (III) peak shifted by +0.36 eV, the C=O peak shifted by +0.8 eV, and the metal oxide peak shifted by +0.68 eV. Conversely, the shift for the gCN phase was negative when comparing the pure gCN to the composite (see Table S1). The observed consistent positive shifts in F@FC binding energies and the negative shifts in gCN binding energies suggest electron migration from the F@FC phase to the gCN phase within the composite. This finding indicates that the Fermi level of F@FC is higher than that of gCN. This finding is consistent with the Mott–Schottky evaluation. According to Mott–Schottky analysis (discussed in more detail in the next chapter; Figure S8), the Fermi levels of F@FC and gCN were determined to be 0.11 V and 1.44 V, respectively. In the F@FC-gCN (1:15) composite, the Fermi level exhibits a decrease to −1.16 V in comparison to the pure gCN, as a result of the F@FC phase incorporation.

### 2.2. Optical and Electrochemical Properties

**Figure 5:**
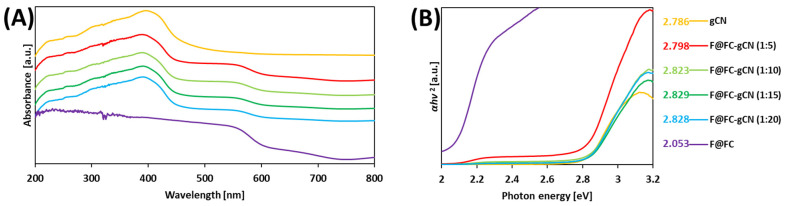
(**A**) UV-Vis diffuse absorbance and (**B**) calculated band gap energies by Tauc plot (Equation (4)).

The optical properties were measured by UV-Vis diffuse spectroscopy (Figure 5A). Yellow-colored bulk gCN absorbs light around the wavelength of 420 nm. On the other hand, red-colored F@FC shows a broad absorption band from the UV region at 200 nm to the Vis region at 570 nm. As illustrated in Figure 5A, the absorption region of the prepared composites extends the spectrum of pure gCN to longer wavelengths due to the optical properties of F@FC. However, as the amount of F@FC in the composite decreases, the intensity of the absorption band at higher wavelengths decreases slightly. The band gap energy (see Figure 5B) was calculated by the Tauc plot method, which involves the following equation:

(4) $${\alpha (h\upsilon )}^{\gamma }=A(\upsilon -E_{g})$$

where α is the absorption coefficient, h is the Planck constant, *υ* is the radiation frequency, A is a proportional constant, E_g_ is the band gap energy, and *γ* is the nature of the electronic transitions with a value of 2 for indirect allowed transitions. At first, both direct and indirect transitions were evaluated during the data analysis. However, as shown in Figure S7, the indirect transition model provides a significantly better fit for this specific system. The band gap energy of pure gCN was determined to be 2.786 eV. The Tauc plot of pure F@FC reveals a distinct, sharp absorption edge at 2.053 eV. Typically, pure CeO_2_ exhibits an E_g_ of approximately 3.0 eV. However, iron doping of the CeO_2_ lattice results in a significant red shift of E_g_ proportional to the iron content [29,31,32]. Alzahrani and Ismail (2023) demonstrated that the incorporation of hematite into a ceria matrix results in a decrease in the band gap energy of the biphasic oxide composite to approximately 2.0 eV at a 20% α-Fe_2_O_3_ loading [32,33,45]. The occurrence of a single sharp absorbance edge for the F@FC material indicates the formation of hierarchically ordered material rather than a simple mechanical mixture of α-Fe_2_O_3_ and Fe-CeO_2_ [46]. The hierarchical arrangement is also observed in F@FC-gCN composites, as evidenced by the occurrence of exclusively one sharp absorption edge. As the amount of F@FC within the composite decreased, a blue shift in band gap energy was observed. The phenomenon of blue-shifted band gap energies in the F@FC-gCN composite originates from electron migration within the material. XPS measurement (conducted in the dark) revealed a clear redistribution of the electron density. The positive shift in the binding energies of cerium, iron, and oxygen for the F@FC phase, and the negative shift for the gCN phase, indicate spontaneous electron migration from the F@FC phase to the gCN within the composite. This influx of electrons into the conduction band of gCN results in a blue shift in the band gap energy of the F@FC-gCN composites. The most substantial blue shift is evident in the F@FC-gCN (1:15) and (1:20) composites (E_g_ of 2.829 and 2.828 eV, respectively). However, as the F@FC content increases, its own low intrinsic band gap energy of 2.023 eV begins to influence the system. This results in a reduction in the blue-shifted band gap to lower values. Despite this decrease, the band gap of F@FC-gCN (1:5) of 2.798 eV remains higher than that of pure gCN, 2.786 eV. This phenomenon is attributable to the sustained charge transfer occurring at the interface.

Mott–Schottky measurements were performed to clarify the photocatalytic mechanism of the investigated samples. This technique relies on applying a bias voltage to modulate the width of the space–charge interfacial double layer and, therefore, the capacitance, assuming the solution side of the interface can be approximated by the Helmholtz model of the double layer [47]. The flat band potentials (V_FB_) were calculated as the intercepts of the extrapolation lines with the potential axis in the Mott–Schottky plot. This plot shows a relationship in which the reciprocal of the measured capacitance squared is plotted against the applied potential. The Mott–Schottky plots for the prepared samples, along with the valence band potentials derived from linear regression analysis within the linear region, are presented in the Supplementary Materials (Figure S8). The negative slope of the approximated line for F@FC signifies its classification as a p-type semiconductor, in contrast to the other samples, which exhibited characteristics typical of n-type semiconductors.

It is assumed that the flat band potential is nearly identical to the conduction band (V_CB_) edge potential according to Equation (5) [48,49].

(5) $$V_{CB}\approx V_{FB\left(NHE\right)}=V_{FB}+∆V-0.059\cdot (7-pH_{0.1MKCl})$$

where V_FB(NHE)_ is the flat band potential recalculated against NHE (normal hydrogen electrode) at pH = 7 and ∆V is the potential of the Ag/AgCl reference electrode vs. NHE of 0.21 V at pH = 7. The valence band edge potential (V_VB_) was calculated using Equation (6) [48,49]

(6) $$V_{VB}=V_{CB}+\frac{E_{g}}{e}$$

where E_g_ is the band gap energy and e is the elementary electron charge.

When a catalyst is irradiated with light of a particular wavelength, electrons are promoted to the conduction band, while holes are formed in the valence band. The electrons and holes then participate in the formation of hydroxyl (•OH) and superoxide (•O_2_^−^) radicals following the reaction pathways (Equations (7)–(9)) [7].

(7) $${OH}^{-}+h^{+}⟶•OH\;\;\left(1.99V\;vs.\;NHE\right)$$

(8) $$O_{2}+e^{-}⟶•O_{2}^{-}\;\;\left(-0.33V\;vs.\;NHE\right)$$

(9) $$H^{+}+•O_{2}^{-}⟶•HO_{2}$$

**Figure 6:**
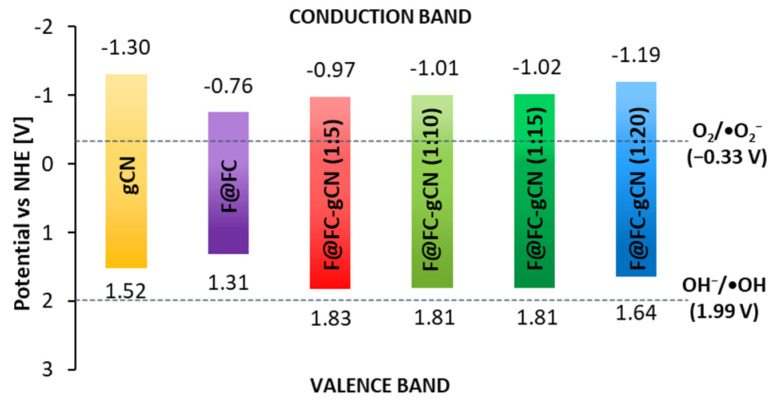
Energy diagram of CB, VB edge potentials, and energy band gaps for the investigated samples.

The conduction and valence band edge potentials and band gap energies are presented in Figure 6. Particularly, V_CB_ was determined to be −1.30 V for pure gCN and −1.02 V for the F@FC-gCN composite (1:15). The conduction band of pure gCN and all prepared F@FC-gCN composites is more negative than the standard O_2_/•O_2_^−^ potential (−0.33 V vs. NHE). This indicates the ability of CB electrons to form superoxide radicals. In contrast, the V_CB_ of pure F@FC was determined to be 0.03 V, which is less than −0.33 V. Hence, the electrons produced by pure F@FC are unable to generate •O_2_^−^ radicals. The valence band edge potentials of pure gCN were determined to be 1.52 V and 1.81 V for the F@FC-gCN composite (1:15). The V_VB_ of pure gCN and all prepared F@FC-gCN composites are considerably lower than the standard OH^−^/•OH redox potential (1.99 V vs. NHE). This suggests that the formation of hydroxyl radicals is improbable. Conversely, for pure F@FC, V_CB_ is 2.10 V, which is higher than the standard OH^−^/•OH redox potential (1.99 V vs. NHE). Hence, the formation of hydroxyl radicals due to the oxidation ability of VB holes is feasible for pure F@FC. The main role of superoxide radicals in photocatalytic degradation is scavenging, as discussed later.

### 2.3. Photocatalytic Degradation Evaluation

**Figure 7:**
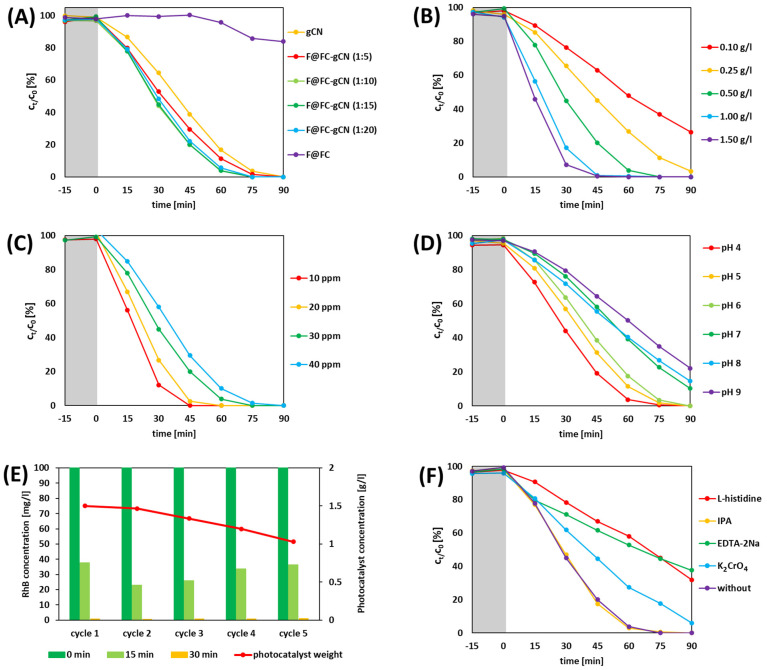
Photocatalytic degradation performance of RhB: (**A**) comparison of the photocatalytic activity of F@FC-gCN composites with pure gCN and F@FC; (**B**) effect of photocatalyst dosage using the F@FC-gCN (1:15) composite; (**C**) influence of the initial RhB concentration on the photocatalytic degradation efficiency using the F@FC-gCN (1:15) composite; (**D**) influence of pH on the photocatalytic degradation using the F@FC-gCN (1:15) composite; (**E**) reusability of the F@FC-gCN (1:15) composite over five consecutive cycles; and (**F**) identification of the dominant reactive species involved in the photocatalytic degradation of RhB using radical scavengers (L-histidine for •O_2_^−^, IPA for •OH, EDTA-2Na for h^+^, and K_2_CrO_4_ for e^−^).

The photocatalytic and Photo-Fenton degradation activity of the prepared photocatalytic composite was studied by evaluating the degradation of Rhodamine B (RhB) as a model water pollutant. The degradation activity of the composites was compared with the activity of pure F@FC and pure gCN. The adsorption of RhB onto the materials’ surface was excluded, as the adsorption–desorption equilibrium was achieved after 30 min in the dark prior to the initiation of irradiation. Initial experiments were conducted at a photocatalyst concentration of 0.5 g/L and an RhB solution concentration of 30 ppm. The photocatalytic degradation of RhB with F@FC material was noticeable after a period of 45 min of irradiation (see Figure 7A). The initial concentration of RhB decreased by a mere 17% by the end of the 90 min experiment. Pure gCN exhibited photocatalytic activity immediately upon irradiation. 

The reaction rate constants for the kinetic experiments were calculated using the pseudo-first-order kinetic model (Equation (10)), which is commonly used for this type of reaction.

(10) $$\ln\left(\frac{c_{0}}{c_{t}}\right)=k\cdot t$$

where c_0_ is the initial concentration, c_t_ is the concentration at time t, and k is the reaction rate constant. The calculated reaction rate constant of degradation with pure gCN was 0.0143 min^−1^ at 30 min of irradiation. The degradation rate of RhB was faster for the prepared F@FC-gCN composites compared to pure gCN. The determined reaction rate constants for the composites were 0.0208, 0.0262, 0.0264, and 0.0234 min^−1^ with increasing amounts of F@FC in composite material (see Table S2 for the summary of reaction rate constants or Figure S10). The accelerated degradation observed in the presence of the composite materials indicates the success of the synthetic process. For further experiments, the composite with the highest degradation activity was selected. Since the reaction rate constants of the F@FC-gCN composite with ratios of 1:10 and 1:15 were very similar, the ratio of 1:15 was selected for the subsequent experiments. This decision was based on the higher specific surface area of the 1:15 composite compared to that of the 1:10 composite.

The effects of environmental parameters on photocatalytic activity were evaluated, including initial pollutant and photocatalyst concentrations, the pH, and the presence of cations and anions. It was observed that RhB degradation accelerated with increasing photocatalyst concentration. For the F@FC gCN composite, the reaction rate constant increased 6.8 times when the amount of photocatalyst increased 10 times. Specifically, 23.6% of 30 ppm RhB was degraded after 30 min with 0.1 g/L of photocatalyst, and 82.9% of RhB was degraded with 1.0 g/L (Figure 7B), respectively. Conversely, an opposing trend was observed in the context of alterations in the initial RhB concentration (Figure 7C). As the initial RhB concentration increased, the reaction rate constant decreased. For instance, the initial concentration of 10 ppm decreased by 87.9% after 30 min of photodegradation using 0.5 g/L of photocatalyst. Meanwhile, the initial concentration of 40 ppm exhibited only a 42.1% decrease.

To investigate the effect of ions, a series of substances was selected for analysis. Specifically, NaCl was used to study the effects of Na^+^ and Cl^−^, NaNO_3_ for NO_3_^−^, Na_2_SO_4_ for SO_4_^2−^, Na_2_CO_3_ for CO_3_^2−^, MgCl_2_ for Mg^2+^, CaCl_2_ for Ca^2+^, and KCl for K^+^. The ions were utilized at a final concentration of 0.01 M in a reaction mixture comprising 30 ppm RhB and 0.5 g/L of photocatalyst. In the presence of inorganic anions (Figure S11A), only a slight decrease in the RhB photocatalytic degradation efficiency was observed for Cl^−^, NO_3_^−^, and SO_4_^2−^. After 45 min of irradiation, 76.8%, 75.3%, and 73.2% of RhB were degraded, respectively, compared to 79.9% in the absence of any anions. This indicates that these anions have only a minor inhibitory effect on the photocatalytic degradation process. The limited influence of Cl^−^, NO_3_^−^, and SO_4_^2−^ anions can be explained by their unfavorable redox potentials relative to the valence band of the F@FC-gCN (1:15) composite, which is approximately 1.81 V vs. NHE. The redox potentials of •Cl/Cl^−^ (2.4 V vs. NHE), •NO_3_/NO_3_^−^ (2.3–2.5 V vs. NHE [50,51]), and •SO_4_^−^/SO_4_^2−^ (2.5–3.1 V vs. NHE [52,53]) are significantly more positive. Consequently, the probability of holes reacting with those anions is low. Therefore, the observed slight decrease in degradation efficiency is more likely attributable to the competitive absorption of anions on the photocatalyst surface [54]. Additionally, the zeta potential shift resulting from electric double layer compression may also influence the degradation effectiveness in the presence of anions [55]. In contrast, CO_3_^2−^ anions strongly suppressed the photocatalytic degradation of RhB. After 45 min of irradiation, only 12.0% of RhB was degraded (k = 0.0027 min^−1^), in contrast to 79.9% (k = 0.0355 min^−1^) observed in the absence of anions. This enhanced inhibition can be attributed to the lower redox potential of •CO_3_^−^/CO_3_^2−^ (1.6–1.8 V vs. NHE [56,57]) compared to the valence band of F@FC-gCN (1:15) (1.81 V vs. NHE). Consequently, carbonate ions can effectively scavenge photogenerated holes, significantly hindering the photocatalytic degradation of RhB.

In the presence of inorganic cations (Figure S11B), only a moderate decrease in RhB photodegradation efficiency was observed compared to the cation-free system. After 45 min of irradiation, 76.9%, 76.8%, 74.4%, and 70.1% of RhB degradation was observed in the presence of Na^+^, K^+^, Mg^2+^, and Ca^2+^ ions, respectively, whereas 79.9% degradation was achieved without any added cations. The inhibitory effect followed the order Na^+^ < K^+^ < Mg^2+^ < Ca^2+^, indicating that divalent cations exert a stronger influence on the degradation process. From an electrochemical perspective, the conduction band potential of the F@FC-gCN (1:15) composite is located approximately −1.02 V vs. NHE. The standard reduction potentials for the examined cations are significantly more negative than the conduction band of the studied photocatalyst, specifically −2.360 V vs. NHE for Mg^2+^/Mg, −2.714 V vs. NHE for Na^+^/Na, −2.838 V vs. NHE for Ca^2+^/Ca, and −2.936 V vs. NHE for K^+^/K [58]. Consequently, these cations cannot be reduced by photogenerated electrons, and direct electron scavenging cannot be a dominant mechanism. However, the presence of cations resulted in a negligible decrease in photocatalytic degradation activity. Similar to anions, this deterioration in degradation efficiency is most likely associated with competitive adsorption of cations on the photocatalyst surface and alterations in the electric double layer, as reflected in changes in zeta potential [55,56].

The impact of pH on the degradation process was examined, as illustrated in Figure 7D. The pH of the RhB solution was adjusted using 0.1 M NaOH and 0.1 M HNO_3_ solutions. The findings demonstrated that under acidic conditions, RhB degradation was most efficient, while at higher pH, the degradation rate decreased. After 45 min of irradiation, 81% of RhB was degraded under pH 4, while only 36% was degraded under pH 9. The F@FC-gCN composite surface exhibited a negative charge (see the zeta potential dependence on zeta potential in Figure S4D), suggesting that electrostatic repulsion might defend the interaction between the photocatalyst and RhB.

The stability of the photocatalyst was evaluated by recycling F@FC-gCN (1:15) for the photocatalytic degradation of 30 ppm RhB, with a starting concentration of a 1.5 g/L photocatalyst. Following the degradation cycle, the photocatalyst was collected by centrifugation and dried at 80 °C for five consecutive photodegradation cycles. It was observed that the photocatalyst’s activity underwent enhancement after the first degradation cycle (see Figure 7E). After 15 min of irradiation, 62%, 77%, and 74% of RhB were degraded in the first, second, and third cycles of the recycling experiment. Further degradation cycles showed a decline in degradation effectiveness (66% and 64% in the fourth and fifth cycles, respectively). However, the amount of reused photocatalyst decreased with each cycle due to the losses during photocatalyst collection after each cycle. Initially, the photocatalyst concentration was 1.5 g/L; however, by the fifth cycle, only 1 g/L of reused photocatalyst remained in the system. It can be estimated that no significant deterioration in photocatalyst efficiency occurs, even after five cycles. The cyclic recovery of cerium ions within F@FC is an important feature of sustainable photocatalytic activity, undergoing a transformation from Ce^3+^ to Ce^4+^ and vice versa during the redox reactions inherent to the degradation system [59,60]. The composite’s structural integrity remained unaltered after five photocatalytic degradation cycles, as evidenced by the TEM images presented in Figure S1C,D,H. According to the results of the XRD, IR, and Raman spectra (see Figure 3A,C,E), no significant differences were observed. The structural aspects of the F@FC-gCN composite exhibited no alteration subsequent to the exposure to the photodegradation process. A comprehensive comparison of the photocatalytic performance of the F@FC-gCN composite with similar carbon nitride-based photocatalysts reported in the recent literature is provided in the Supplementary Materials (Table S4).

### 2.4. Photo-Fenton Degradation Evaluation

The Photo-Fenton degradation of Rhodamine B was studied under the same conditions as for photocatalytic degradation, but with hydrogen peroxide added in advance. During the Photo-Fenton degradation, the F@FC-gCN (1:10) and (1:15) composites exhibited the highest degradation rates. These two composites exhibited the highest degradation rate, both in photocatalytic and Photo-Fenton degradation.

**Figure 8:**
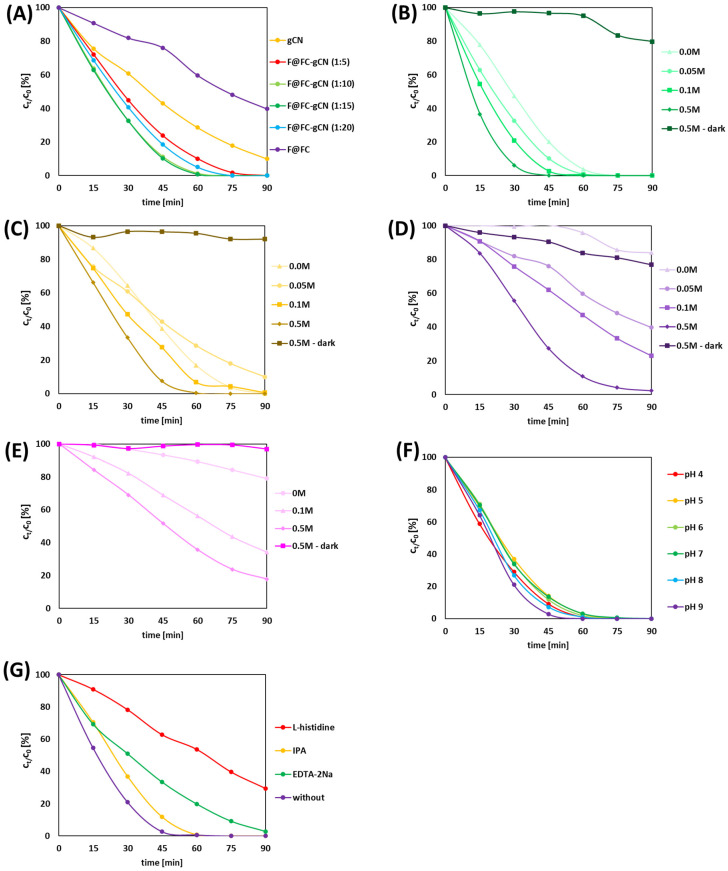
Photo-Fenton degradation of Rhodamine B using hydrogen peroxide. (**A**) Comparison of the Photo-Fenton degradation activity of F@FC-gCN composites with pure gCN and F@FC using a final H_2_O_2_ concentration of 0.05 M. The influence of H_2_O_2_ concentration on RhB Photo-Fenton degradation using (**B**) the F@FC-gCN (1:15) composite, (**C**) pure gCN, and (**D**) pure F@FC. (**E**) Photolysis of RhB under various conditions. (**F**) Influence of pH on the Photo-Fenton degradation using the F@FC-gCN (1:15) composite at a fixed H_2_O_2_ concentration of 0.05 M. (**G**) Identification of the dominant reactive species involved in the photocatalytic degradation of RhB using radical scavengers (L-histidine for •O_2_^−^, IPA for •OH, and EDTA-2Na for h^+^).

The prepared F@FC-gCN (1:15) composite demonstrated a 1.4 times higher reaction rate constant in comparison to Photo-Fenton degradation activity (with 0.05 M H_2_O_2_), with photocatalytic degradation activity following 45 min of irradiation (Figure 8A compared to Figure 7A; see also Tables S2 and S3 in the Supplementary Materials for a comprehensive summary of reaction rate constants or Figure S10). Conversely, the presence of H_2_O_2_ in the Photo-Fenton degradation has been shown to inhibit the degradation of RhB with pure gCN by approximately 0.9 times after 45 min of irradiation. Pure F@FC demonstrated high Photo-Fenton activity, exhibiting 22 times higher activity than photocatalytic degradation after 60 min of irradiation. This observation indicates that the presence of F@FC predominantly enhances the Photo-Fenton degradation activity of F@FC-gCN (1:15) composite.

Moreover, the F@FC-gCN (1:15) composite exhibits Fenton degradation activity. In the initial 60 min of the Fenton degradation experiment (final H_2_O_2_ concentration of 0.5 M in the degradation experiment, and conducted under dark conditions), it appears that slight adsorption of RhB primarily occurs on the surface of both the F@FC-gCN (1:15) composite and pure gCN, as evidenced in Figure 8B,C. After a duration of 90 min, 20.2% of RhB was degraded using the F@FC-gCN (1:15) composite, while 7.9% was degraded using pure gCN. Pure F@FC exhibited Fenton activity itself from the initiation of Fenton degradation (see Figure 8D), with 23.1% of RhB degraded after 90 min. This observation indicates that F@FC enables the Fenton activity of the F@FC-gCN composite. The initiation of Fenton degradation of the F@FC-gCN (1:15) composite is inhibited in comparison to pure F@FC, likely attributable to the reduced F@FC content in this F@FC-gCN (1:15) composite.

It was observed that degradation of RhB tended to accelerate with increasing concentration of hydrogen peroxide in the system (range 0.05 to 0.5 M H_2_O_2_) for the F@FC-gCN (1:15) composite (Figure 8B) and pure gCN (Figure 8C), as well as pure F@FC (Figure 8D). Moreover, it is noteworthy that photolysis of RhB occurred (see Figure 8E).

An important finding was the distinct influence of pH on the photocatalytic and Photo-Fenton degradation of the F@FC@gCN (1:15) composite. The photocatalytic degradation activity of this material exhibits remarkable efficiency only in an acidic environment. Conversely, the Photo-Fenton degradation activity was revealed to be pH independent, with consistent degradation activity of RhB across the entire pH range tested (Figure 8F). As demonstrated in Figure S12, the pH independence of Photo-Fenton degradation is not influenced by the H_2_O_2_ concentration. The ability of the prepared F@FC-gCN photocatalyst to degrade RhB using hydrogen peroxide in Photo-Fenton degradation across the entire pH range is crucial for its practical application in environmental remediation. The homogeneous Photo-Fenton degradation activity is generally most effective at acidic pH, due to the prevention of the precipitation of free Fe^3+^ ions. Oppositely, the F@FC-gCN system exhibits minimal variation across the entire pH range. This is attributed to the stable incorporation of iron into the hematite lattice, as well as into the CeO_2_ lattice via doping. According to the results, the Photo-Fenton degradation efficiency is slightly higher at alkaline pH due to the increased concentration of OH^−^ ions. The Ce^3+^/Ce^4+^ redox system, induced by iron doping in the CeO_2_ crystal lattice, may also partially contribute to this stable activity within the entire pH range [61].

### 2.5. Possible Photocatalytic and Photo-Fenton Degradation Mechanism

Scavenging experiments were performed to understand the generation of active species by the F@FC-gCN (1:15) composite and thus determine the degradation mechanism of this photocatalyst within both photocatalytic and Photo-Fenton degradation processes. EDTA-2Na (ethylenediaminetetraacetic acid disodium salt dihydrate), K_2_CrO_4_, IPA (isopropanol), and L-histidine were used as h^+^, e^−^, •OH, and •O_2_^−^ scavengers. The scavenging experiments were performed with a photocatalyst concentration of 0.5 g/L. All scavengers were utilized at a concentration of 0.01 M within the 30 ppm RhB solution.

The investigation revealed that IPA exhibited no significant influence on the RhB photocatalytic degradation, as evidenced in Figure 7F. Consequently, it can be concluded that •OH did not contribute to the photocatalytic degradation process. In contrast, when the reaction was scavenged by L-histidine, EDTA-2Na, and K_2_CrO_4_, the photocatalytic degradation of RhB decreased from 79.9% of degraded RhB in 45 min to 33.0%, 38.4%, and 55.4%, respectively. These results indicate that •O_2_^−^, h^+^, and e^−^ play a role in photocatalytic degradation of RhB using the F@FC-gCN composite. However, among the investigated reactive species, only •O_2_^−^ plays a dominant role at the initial stage of photocatalytic degradation. This is evidenced by the suppression of RhB degradation in the presence of L-histidine. After 15 min of irradiation, only 9.3% of RhB was degraded when L-histidine was used to scavenge the degradation products, compared to approximately 20% with other scavengers. This finding indicates a significant impact of •O_2_^−^ on the photocatalytic degradation process from the beginning of the degradation. The outcomes of the scavenging experiments are consistent with the Mott–Schottky measurements. Particularly, the valence band potential for the F@FC-gCN (1:15) composite was established as −1.02 V vs. NHE, which exceeds the standard redox potential for O_2_/•O_2_^−^ (−0.33 V vs. NHE). Conversely, the conduction band potential of this photocatalyst (1.81 V vs. NHE) is considerably lower than the standard redox potential for OH^−^/•OH (1.99 V vs. NHE), thereby hindering the generation of hydroxyl radicals.

Similarly, the Photo-Fenton degradation mechanism of the F@FC-gCN (1:15) composite was proposed (see Figure 8G). The scavenging of •OH by IPA decreased the degradation rate. The presence of H_2_O_2_ leads to the formation of hydroxyl radicals (see Equation (1)). •OH generation was observed already at the initial stage of Photo-Fenton degradation. Compared to the photocatalytic degradation mechanism, the generation of •OH was not observed at all. In the Photo-Fenton degradation process, the roles of h^+^ and •O_2_^−^ are analogous to the photocatalytic degradation, underscoring the significance of these species in the overall reaction mechanism. According to L-histidine scavenging degradation, it was observed that RhB was degraded by 68% within the photocatalytic approach (Figure 7E) and 71% within the Photo-Fenton degradation approach (Figure 8G) within 90 min of irradiation. Therefore, the production of •O_2_^−^ primarily occurred due to the photocatalytic properties of the composite, rather than the impact of H_2_O_2_. The scavenging of e^−^ was not performed to study the Photo-Fenton degradation process, since K_2_CrO_4_ is immediately reduced by hydrogen peroxide itself.

### 2.6. Possible Degradation Pathways

**Figure 9:**
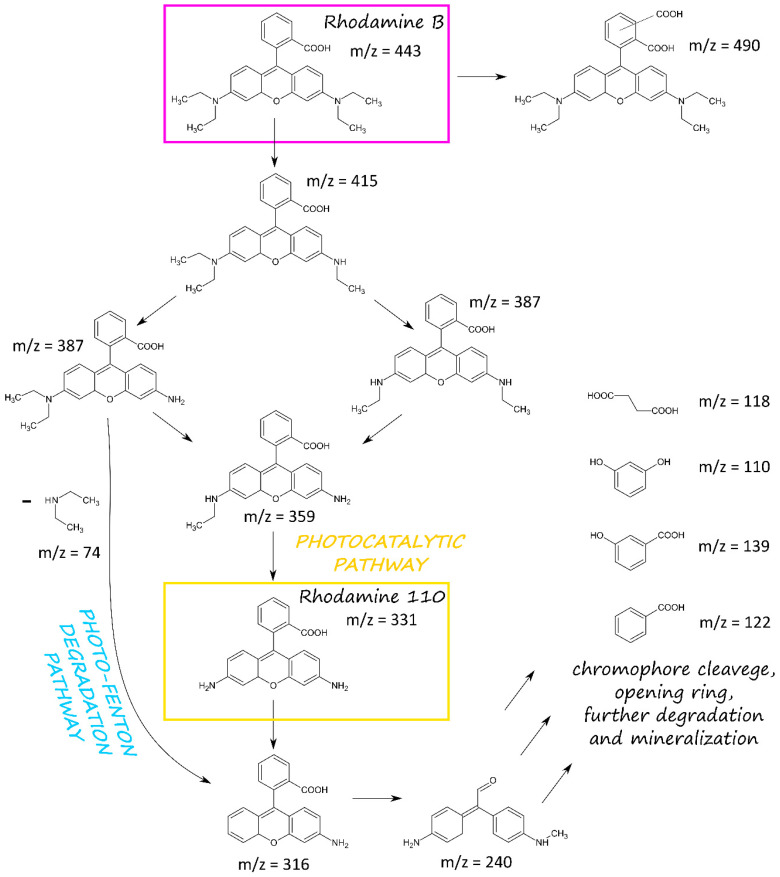
Differences in the possible degradation pathway for photocatalytic and Photo-Fenton degradation using the F@FC-gCN (1:15) composite.

Since a visible difference between photocatalytic degradation and the Photo-Fenton process was observed during the degradation experiments (see Figure S9), HPLC-MS (High-Performance Liquid Chromatography–Mass Spectrometry) was used to identify RhB degradation intermediates. Based on previous studies [62,63,64,65], the degradation pathway of RhB was proposed (Figure 9). In both processes, photocatalytic and Photo-Fenton degradation, N-deethylation occurred first. Initially, *m*/*z* = 443 and 415 with 97% and 3% ratios as Rhodamine B and N,N-diethyl-N′-ethylrhodamine were observed in the HPLC-MS spectrum. After applying light, thereby initiating the photodegradation process, N-de-ethylation begins, giving the formation of intermediates with *m*/*z* = 387 and 359, corresponding to N,N-diethylrhodamine, N-ethyl-N′-ethylrhodamine, and N-ethylrhodamine. During photocatalytic degradation, most of these intermediates form Rhodamine 110, which exhibits characteristic green-yellow luminescence (Figure S9) [66]. Conversely, no signal of Rhodamine 110 with *m*/*z* = 331 was detected during the Photo-Fenton degradation pathway. Instead, *m*/*z* = 316 was observed by eliminating diethylamine (*m*/*z* = 74) from RhB and its intermediates. According to Jakimińska et al. (2022), the conversion of RhB to Rhodamine 110 occurs under low concentrations of reactive oxygen species [66]. This observation is consistent with the presented results, as no •OH were produced during the photocatalytic degradation approach. However, •OH was clearly detected during the Photo-Fenton degradation. Consequently, the generated •OH likely prevents the conversion of Rhodamine B to Rhodamine 110. A deaminated intermediate with *m*/*z* = 316 was observed following 30 min of irradiation for the Photo-Fenton pathway and after 75 min for the photocatalytic degradation. Chromophore cleavage, ring opening, and further degradation were observed at *m*/*z* values below 300, following completion of N-deethylation.

## 3. Materials and Methods

### 3.1. Chemicals for Composite Synthesis

Melamine, cerium(III) nitrate hexahydrate, and 20% tetraethylammonium hydroxide solution were purchased from Sigma-Aldrich (St. Louis, MO, USA). Iron(III) nitrate nonahydrate, oxalic acid, and sodium hydroxide were purchased from PENTA (Prague, Czechia). Methanol was purchased from Lach-Ner (Neratovice, Czechia). All used chemicals were of p.a. quality.

### 3.2. Synthesis of Photocatalyst

**Scheme 1:**
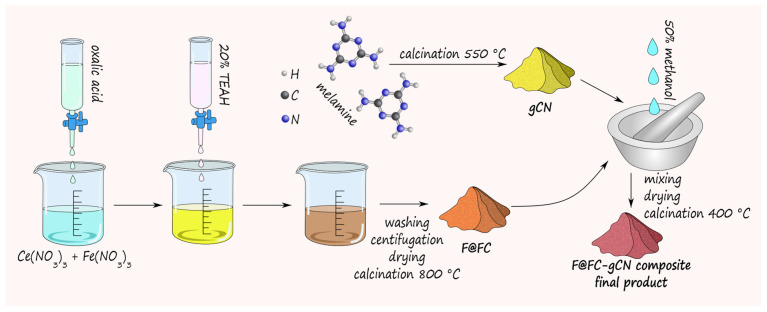
A schematic diagram illustrating the synthetic process of hematite decorated with iron-doped cerium oxide and carbon nitride composite.

The synthesis of α-Fe_2_O_3_/Fe-CeO_2_ (F@FC), graphitic carbon nitride (gCN), and their hierarchically structured composites are illustrated in Scheme 1.

A total of 4.34 g of cerium(III) nitrate hexahydrate and 4.04 g of iron(III) nitrate nonahydrate were dissolved in 100 mL of distilled water. A total of 100 mL of 0.2 M oxalic acid solution was added dropwise to the mixture. Subsequently, 20 mL of 20% tetraethylammonium hydroxide (TEAH) solution was added dropwise, during which a yellow solution formed. To the yellow solution, 5 M NaOH was added until the pH reached 9. A light-brown precipitate was obtained and stirred for an hour. Then, the precipitate solution was centrifuged, washed several times with distilled water, dried at 80 °C, and finally calcined at 800 °C for 4 h with a heating rate of 4.5 °C/min under an air atmosphere.

The gCN was synthesized by calcining 10 g of melamine in a muffle furnace at 550 °C for 4 h under air, with a heating rate of 3 °C/min. After cooling, the product was ground in a porcelain mortar into a powder.

Prepared F@FC and gCN powders were mixed in a porcelain mortar at ratios of F@FC to gCN of 1:5, 1:10, 1:15, and 1:20 with a 50% methanol solution in water and dried at 90 °C. The stirring and drying process was repeated three times overall. Subsequently, the prepared composite was annealed at 400 °C for 2 h with a heating rate of 6.5 °C/min in an air atmosphere to ensure the formation of an active interphase between the individual components of the composite. The resulting material was labeled as F@FC-gCN (1:X), where X means the ratio used during preparation.

### 3.3. Characterization of Photocatalyst

The morphology of the synthesized material was examined using a transmission electron microscope (TEM) JEM-2010 (JEOL, Tokyo, Japan), a scanning electron microscope (SEM) SU-6600 (Hitachi, Tokyo, Japan), and a high-resolution transmission electron microscope (HRTEM) Titan 60–300 kV (FEI, now Thermo Fisher Scientific, Wltham, MA, USA). The structural composition of the prepared samples was analyzed by X-ray powder diffraction (XRD) using the Aeris benchtop diffraction system (Malvern Panalytical, Malver, UK). The XPS measurements were performed using the Nexsa G2 XPS system (Thermo Fisher Scientific, Waltham, MA, USA). Infrared spectroscopy was performed on a Nicolet iS50 FT-IR Spectrometer (Thermo Fisher Scientific, Waltham, MA, USA) in ATR mode with a diamond crystal. The Raman spectroscopy was measured using a DXR3 Raman microscope (Thermo Fisher Scientific, Waltham, MA, USA). The specific surface area and pore volumes were measured using a gas sorption analyzer, 3Flex (Micrometrics, Norcross, GA, USA). The zeta potential was measured using a Zetasizer Nano Series equipped with an MPT-2 multipurpose titrator (Malvern Pananalytical, Malvern, UK). UV-Vis absorption and diffuse reflectance spectra were measured with a UV-Vis spectrometer SPECORD PLUS 250 with an integrating sphere (Analytik Jena, Jena, Germany). The electrochemical tests were conducted using a Metrohm Autolab PGSTAT302 potentiostat (Metrohm, Utrecht, The Netherlands), with the glassy carbon electrode (GCE) as the working electrode, and an Ag/AgCl (3 M KCl) electrode and a platinum sheet as the reference and counter electrodes, respectively. The Supplementary Materials provide a thorough description of all measurement conditions associated with the characterization methods.

### 3.4. Photodegradation Evaluation

The photocatalytic activity was evaluated by the degradation of Rhodamine B (RhB). In the standard procedure, 0.5 g/L of the photocatalyst was used to degrade 20 mL of a RhB solution at 30 ppm. The degradation was performed in the Photocube (ThalesNano, Budapest, Hungary) photoreactor under LED white light (5920 lm, 1500 mA, color temperature of 6500 K) with the air-cooling system and a stirring rate of 720 rpm. The degradation was initiated for 30 min in the dark to reach adsorption–desorption equilibrium. Sampling was done every 15 min by removing 0.75 mL of sample and diluting it with 0.75 mL of distilled water. The diluted sample was centrifuged and then transferred to the vial for HPLC analysis.

The standard procedure was changed to 0.1, 0.25, 1.0, and 1.5 g/L of photocatalyst and 10, 20, and 40 ppm of RhB solution to evaluate photocatalyst activity and optimize degradation conditions. To analyze the influence of pH, the pH of the RhB stock solution was adjusted by 0.1 M NaOH or HNO_3_. The influence of ions was established using the final ion concentration of 0.01 M in the 30 ppm RhB solution.

The Photo-Fenton degradation was evaluated by adding hydrogen peroxide solutions with varying final concentrations (0.05, 0.1, and 0.5 M) to a RhB solution, resulting in a final RhB concentration of 30 ppm. The photocatalyst concentration used was 0.5 g/L. The irradiation in the Photocube (ThalesNano, Budapest, Hungary) photoreactor under LED white light started immediately following the addition of hydrogen peroxide.

High-performance liquid chromatography (HPLC) was performed to determine RhB concentration after photocatalytic experiments. The analysis was performed on an LC Agilent 1260 Infinity II (Agilent, Santa Clara, CA, USA) with a Kinetex C_18_ column 150 × 4.6 mm (Phenomenex, Torrace, CA, USA) packed with 5 µm particle size. The mobile phase consisted of 0.1% H_3_PO_4_ and acetonitrile in a 50:50 (*v*/*v*) ratio. The analysis was performed at a constant flow rate of 1 mL/min and an injection volume of 20 µL. Rhodamine B was detected at a wavelength of 554 nm. Data analysis was processed using OpenLab CDS 3.5 software (Agilent, Santa Clara, CA, USA) with automatic integration.

## 4. Conclusions

The hierarchically structured hematite phase decorated with iron-doped cerium oxide nanoparticles and its composites with graphitic carbon nitride (F@FC-gCN), synthesized in this study, exhibited high efficacy for both photocatalytic and Photo-Fenton degradation of the model pollutant Rhodamine B. Based on catalyst characterization and primary degradation tests, the composite ratios of 1:10 and 1:15 were determined to be optimal. The degradation efficiency of the F@FC-gCN composite was found to be two times higher than pure gCN for photocatalysis and four times higher for Photo-Fenton degradation within 60 min of irradiation. The most important finding of this study was the observed independence of the Photo-Fenton degradation activity from pH, using the F@FC-gCN composite as a photocatalyst. This phenomenon is likely related to the stable incorporation of iron into the hematite lattice, as well as into the CeO_2_ lattice via doping. This finding offers a substantial advantage for photodegradation in real applications, as the F@FC-gCN composite can degrade pollutants, even under neutral conditions, which are typical of natural water resources. Moreover, the cyclic recovery of the F@FC-gCN composite demonstrates high recyclability, as the material exhibits retained degradation effectiveness. This may be attributed to the self-cleaning effect induced by the Ce^3+^/Ce^4+^ redox pair, which is enabled by oxygen vacancies in the CeO_2_ lattice resulting from iron doping.

## Data Availability

All data are included in the text.

## Supplementary Materials

The following supporting information can be downloaded at: https://www.mdpi.com/article/10.3390/ijms27073133/s1 [67,68,69,70,71].
