# Supplementary material for: Photocatalytic and Photo-Fenton Degradation Activity of Hierarchically Structured α-Fe2O3@Fe-CeO2 and g-C3N4 Composite
Source: Int J Mol Sci. 2026 Mar 30;27(7):3133. doi: 10.3390/ijms27073133 (PMC13073559; doi:10.3390/ijms27073133)
Supplement: Supplementary file 1 [file ijms-27-03133-s001.zip › ijms-4155391-supplementary.pdf]

Support Material

Photocatalytic and Photo Fenton degradation activity of hierarchically structured α-Fe_2O_3@Fe-CeO_2 and g-C_3N_4 composite

Aneta Bužková[1], Radka Pocklanová[1]*, Vlastimil Novák[2], Martin Petr[3], Barbora Štefková[1], Alexandra Rancová[1], Josef Kašlík[3], Robert Prucek[1], Aleš Panáček[1], Libor Kvítek[1]*

[1] Department of Physical Chemistry, Faculty of Science, Palacký University, 17. listopadu 1192/12, 779 00 Olomouc, Czech Republic

[2] Department of Chemistry and Physico-Chemical Processes, Faculty of Materials Science and Technology, Technical University of Ostrava, 17. listopadu 2172/15, 708 00 Ostrava-Poruba, Czech Republic

[3] Regional Centre of Advanced Technologies and Materials (RCPTM), Czech Advanced Technology and Research Institute (CATRIN), Palacký University, Šlechtitelů 241/27, 779 00 Olomouc, Czech Republic

*Corresponding author

e-mail: radka.pocklanova@upol.cz (R. Pocklanová)

libor.kvitek@upol.cz (L. Kvítek)

1. Characterization

The morphology of synthesized material was examined by a transmission electron microscope JEM 2010 (JEOL), a scanning electron microscopy SU 6600 (Hitachi), and a high-resolution transmission electron microscope Titan 60-300 kV (FEI). The structural composition of prepared samples was analyzed by X-ray powder diffraction (XRD) using Aeris benchtop diffraction system (Malvern, Panalytical) operating in Bragg-Brentano geometry, equipped with iron filtered CoKα radiation source. The angular range of measurement was from 5 to 105° 2θ. The data were processed using High Score Plus software in conjunction with PDF and ICSD databases.

The XPS measurements were carried out with the Nexsa G2 XPS system (Thermo Fisher Scientific) with a monochromatic Al-Kα source and photon energy of 1486.7 eV. All the spectra were acquired in the vacuum of 1.2 · 10-7 Pa and at the room temperature of 20 °C. The analyzed area on each sample was spot of 200 μm in diameter. The survey spectra were measured with a pass energy of 150.00 eV and electronvolt step of 1.0 eV, while for the high-resolution spectra were used with a pass energy of 30.00 eV and electronvolt step of 0.1 eV. Charge compensation was used for all measurements. The spectra were evaluated with the Avantage 6.5.1 (Thermo Fisher Scientific) software. Infrared spectroscopy was performed by Nicolet iS50 FT IR Spectrometer (Thermo Fisher Scientific) in ATR configuration with diamond crystal. The Raman spectroscopy was measured using DXR3 Raman microscope (Thermo Fisher Scientific) equipped with 785 nm laser (30 mW for pure gCN, 5 mW for the rest of samples), 900 lines/mm grating, 25 μm pinhole aperture 10x/0.25 NA objective. 50 exposures for both sample (with fluorescence correction – polynomial order 3) and background was proceeded. The specific surface area and pore volumes were collected by gas sorption analyzer 3Flex (Micrometrics). The amount of sample from 0.1 to 0.2 g (except CFO 0.8 g) was degassed at 200 °C for 2 hours, followed by 130 °C for 12 hours. Measurement started by evacuation of the sample, continued by physisorption/desorption of N_2 in the liquid N_2 bath and subsequent measurement of the free space using helium. The evaluation was proceeded by standard BET method with the fitting of BET surface area plot using Rouquerol function. The zeta potential was measured by Zetasizer Nano Series (Malvern) equipped with MPT-2 multipurpose titrator (Malvern) using 0.1M HNO_3, 0.1M NaOH, and 0.05M NaOH water solutions for automatic pH adjustment.

UV-Vis absorption and diffuse reflectance spectra were measured with UV-Vis spectrometer SPECORD PLUS 250 with an integrating sphere (Analytik Jena).

Electrochemical measurement was proceeded as follows. Samples weighing 10 mg were added to 5 ml of deionized water to create a suspension, which was then homogenized using an

ultrasonicator for 30 minutes. Following this, 30 µl of the suspension was deposited onto the surface of a glassy carbon electrode, which was subsequently dried at 85 °C for 3 hours. The electrochemical tests were conducted using a Metrohm Autolab PGSTAT302 potentiostat, with the glassy carbon electrode (GCE) serving as the working electrode, while an Ag/AgCl (3M KCl) electrode and a platinum sheet were utilized as the reference and counter electrodes, respectively. Before each measurement, the surface of the glassy carbon electrode was cleaned using a polishing set designed for solid-state electrodes (Metrohm). Electrochemical measurements were carried out in a 0.1 M KCl solution previously bubbled with nitrogen for 30 min. Mott-Schottky measurements were executed at an alternating frequency of 300 Hz with an amplitude of 10 mV. The degradation pathway of Rhodamine B was evaluated by UPLC-MS analysis performed on Acquity H-class UPLC chromatography with PDA and MS detector (Waters) with C_{18} column C-Select HSS T3 (2.5 µm, 3.0 mm × 50 mm) (Waters) and with guard column (Waters). The column temperature was maintained at 30 °C. The mobile phase consisted of (A) 0.01 M ammonium acetate in water and (B) acetonitrile. The analysis proceeded with a gradient elution (Fast gradient 50-80-80% B in A; 0-3-5 min) and a constant flow rate of 0.6 ml/min. The ESI source was operated at a capillary voltage of 3 kV, with a desolvation temperature of 350 °C and a source temperature of 120 °C.

2. Results

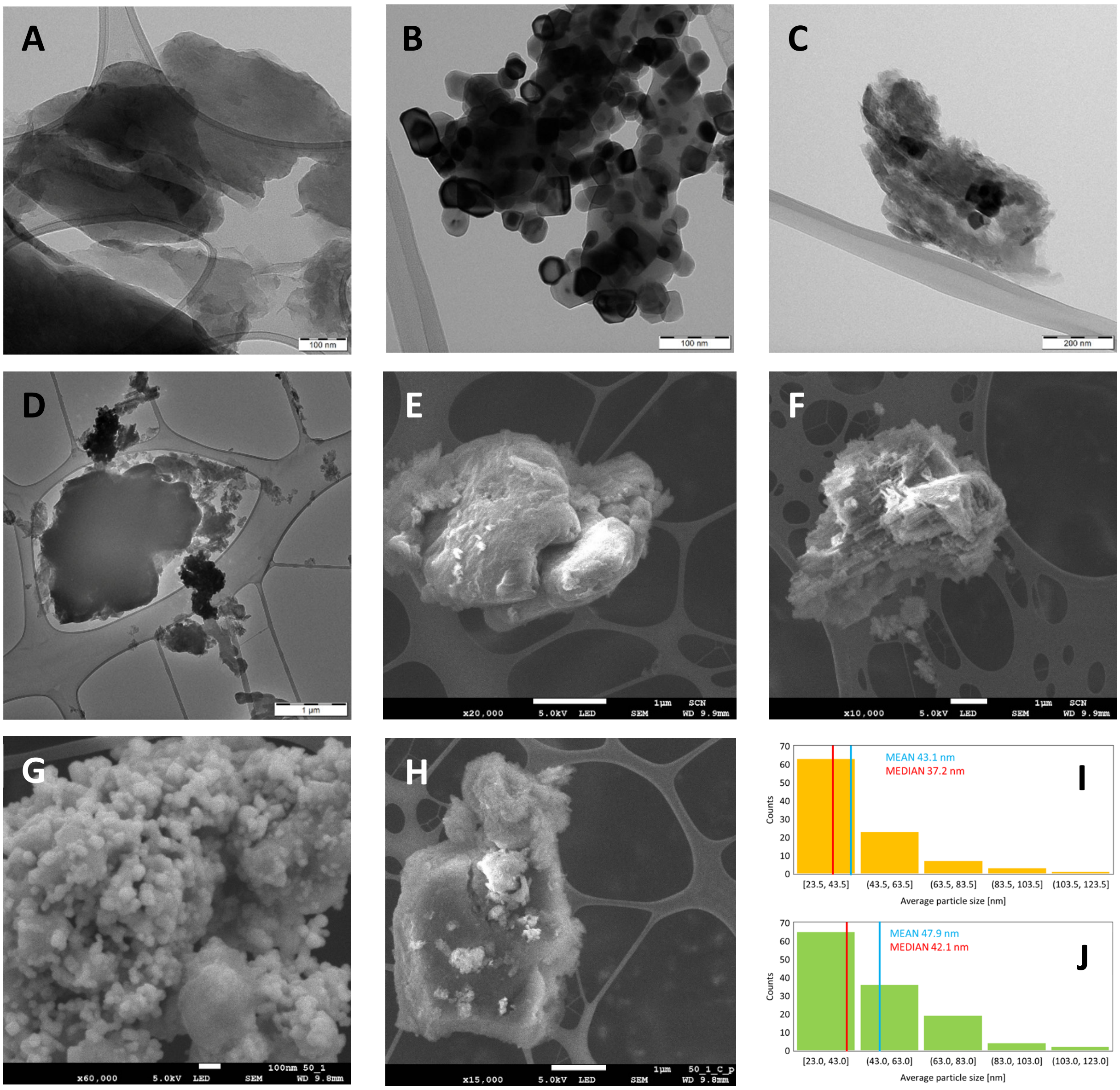

Fig. S1: TEM images of **(A)** pure gCN, **(B)** pure F@FC, and **(C, D)** F@FC-gCN (1:15) nanocomposite after five photodegradation cycles. SEM images of **(E, F)** pure gCN, **(G)** pure F@FC, and **(H)** F@FC-gCN (1:15) nanocomposite after five photodegradation cycles. Average particle size of the CeO_2 nanoparticles in the pure F@FC sample counted from **(I)** TEM image (Fig. S1B), and **(J)** SEM image (Fig. S1G) using Digimizer software.

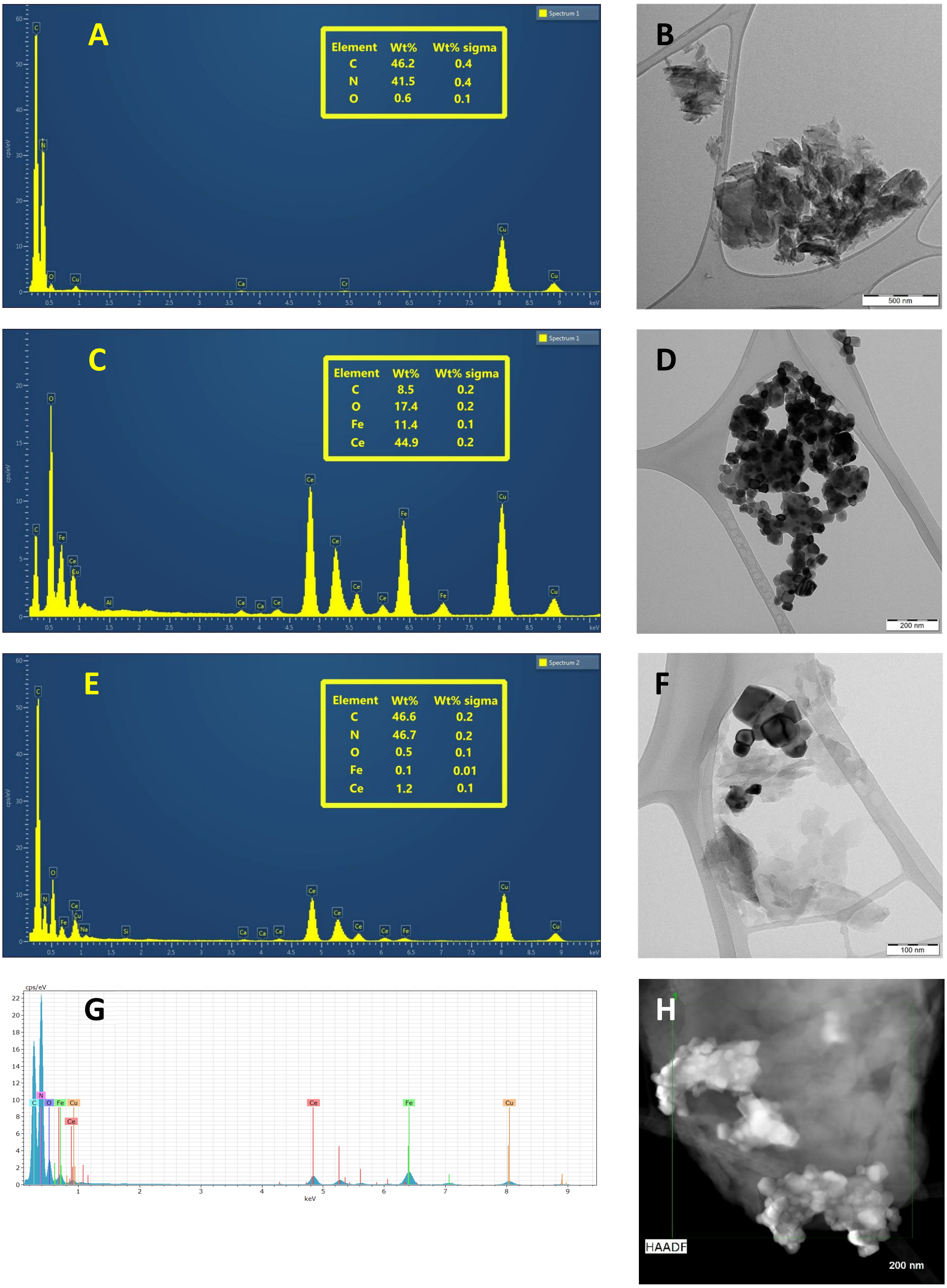

Fig. S2: EDS spectrum with corresponding TEM images for **(A, B)** pure gCN, **(C, D)** pure F@FC, and **(E, F)** nanocomposite material F@FC-gCN with a ratio of 1:15. **(G)** EDS spectrum taken by HRTEM, and **(H)** the corresponding area of this spectrum in HAADF imaging mode.

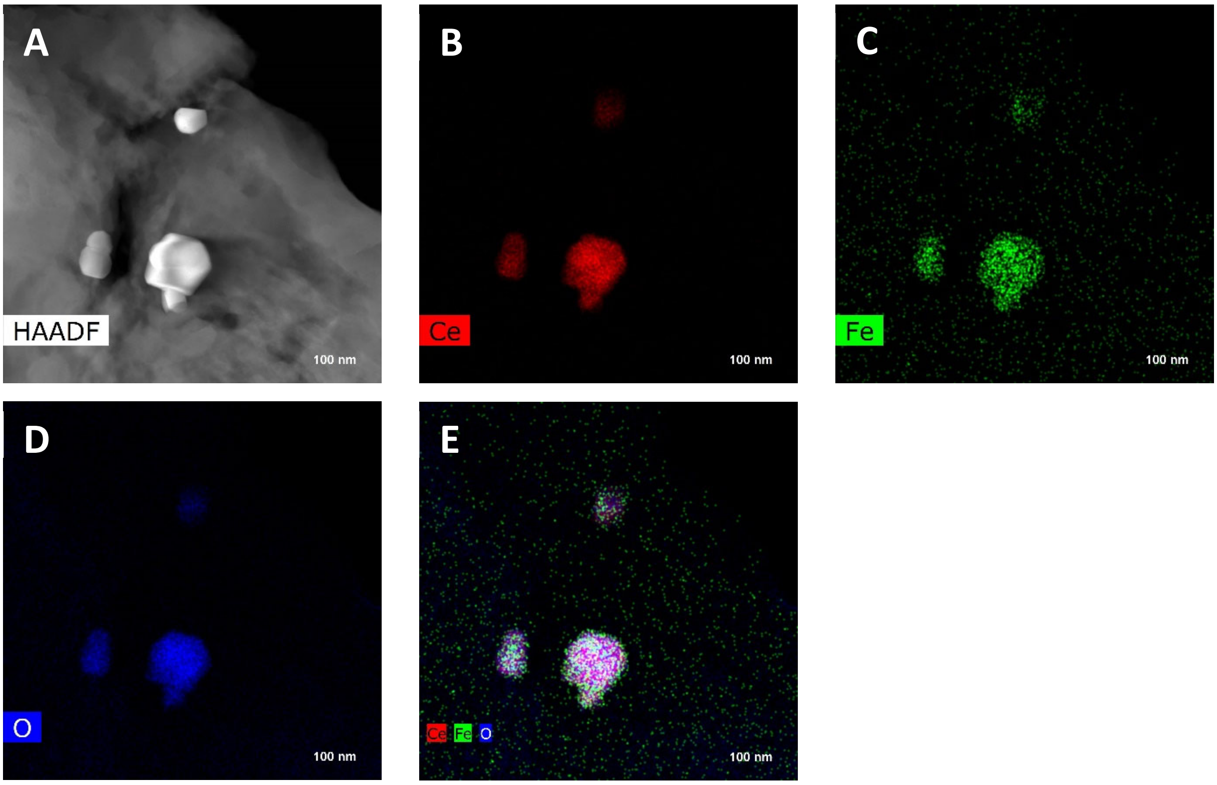

Fig. S3: (A) HAADF image and elemental mapping of **(B)** cerium, **(C)** iron, **(D)** carbon and nitrogen, **(E)** cerium, iron and oxygen, of F@FC-gCN (1:15) nanocomposite focused to single isolated nanoparticle.

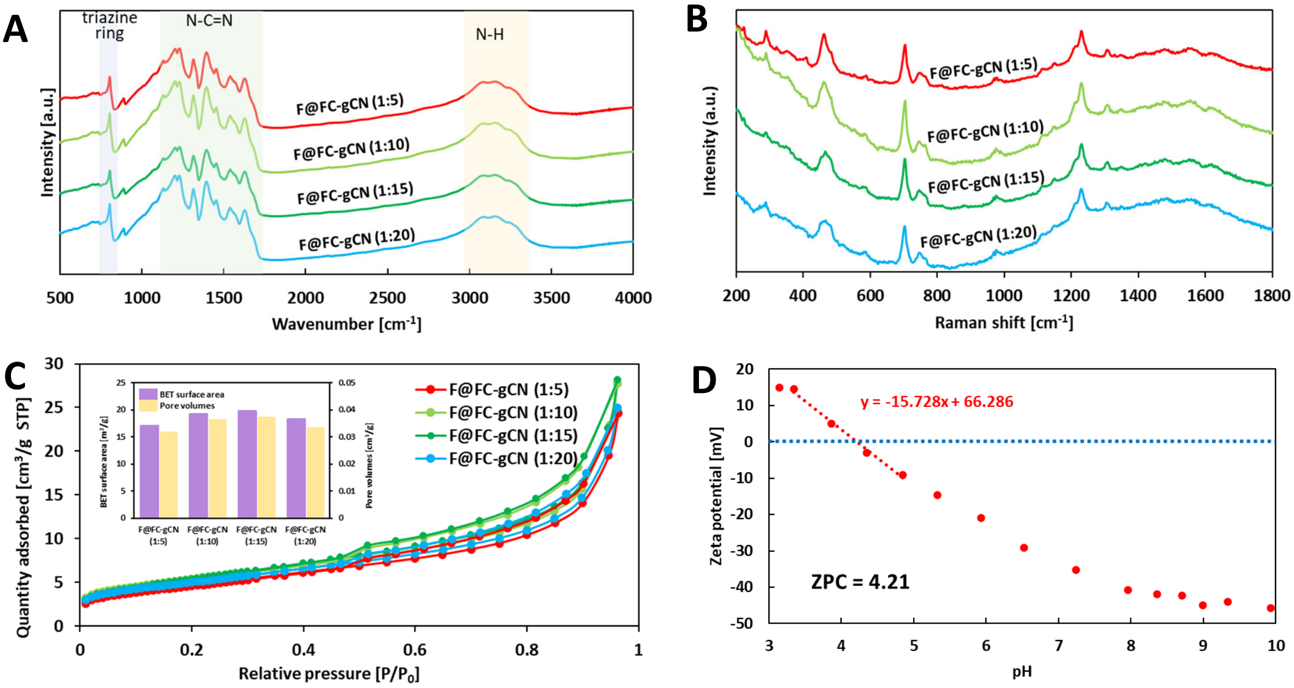

Fig. S4: (A) The IR spectra of the F@FC-gCN composites in different ratios. **(B)** The Raman spectra. **(C)** N_2 adsorption-desorption isotherms with pore volumes and BET specific surface area. **(D)** Influence of pH on the zeta potential of F@FC-gCN (1:15) composite.

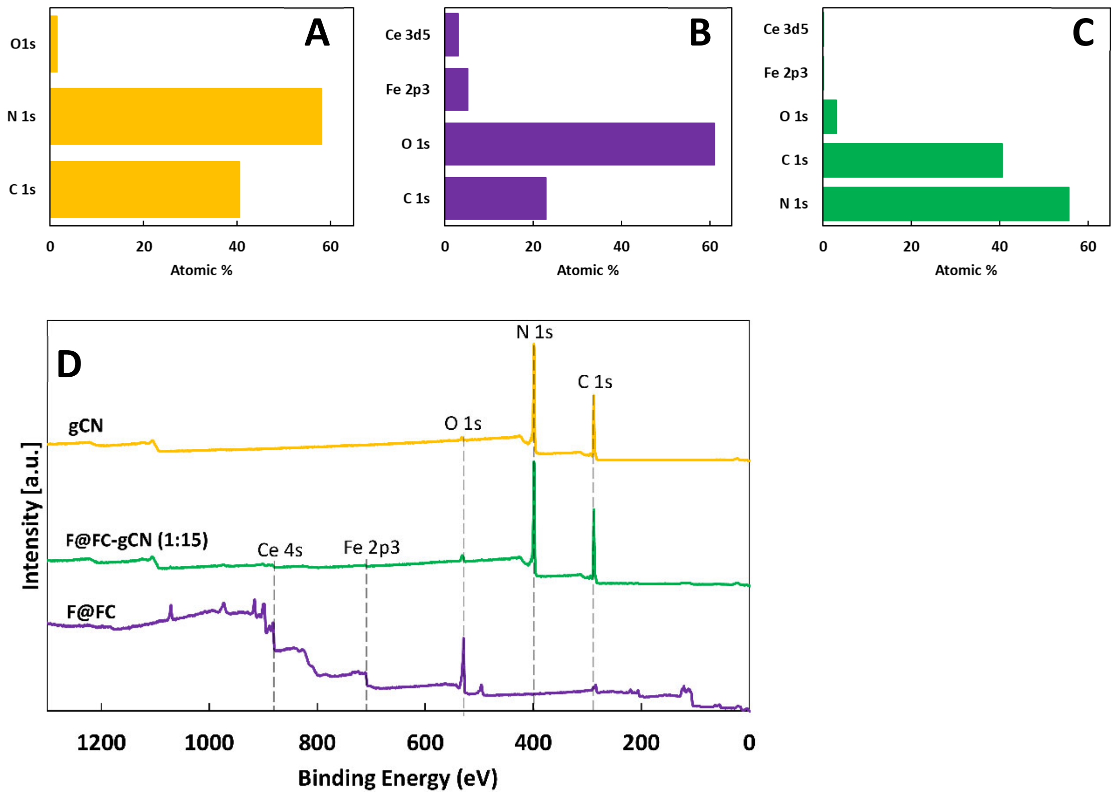

Fig. S5: Atomic percentage of **(A)** gCN, **(B)** F@FC, and **(C)** F@FC-gCN (1:15) composite determined by XPS measurement, and **(D)** XPS survey spectra of pure gCN, pure F@FC and F@FC-gCN composite (1:15).

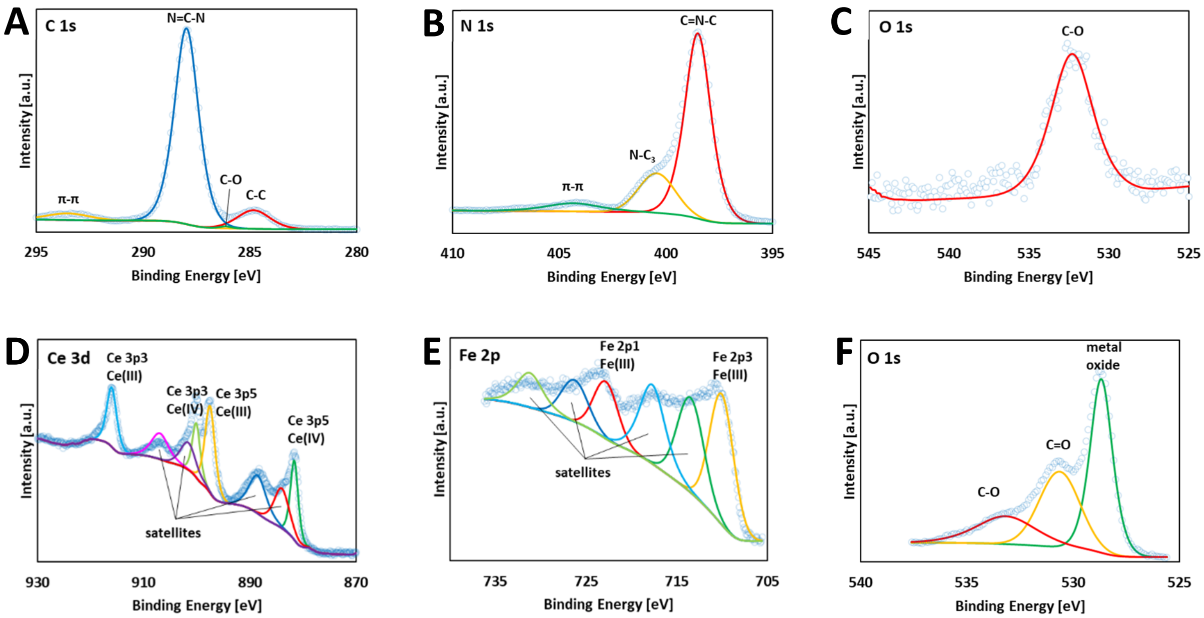

Fig. S6: The XPS spectra deconvolution of the gCN material **(A)** C 1s, **(B)** N 1s, **(C)** O 1s, and F@FC material **(D)** Ce 3d, **(E)** Fe 2p, and **(F)** O 1s.

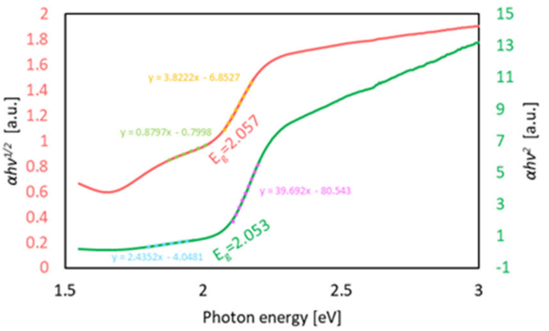

Fig. S7: The Tauc plot and calculated band gap energies of pure F@FC sample for direct and indirect electronic transitions.

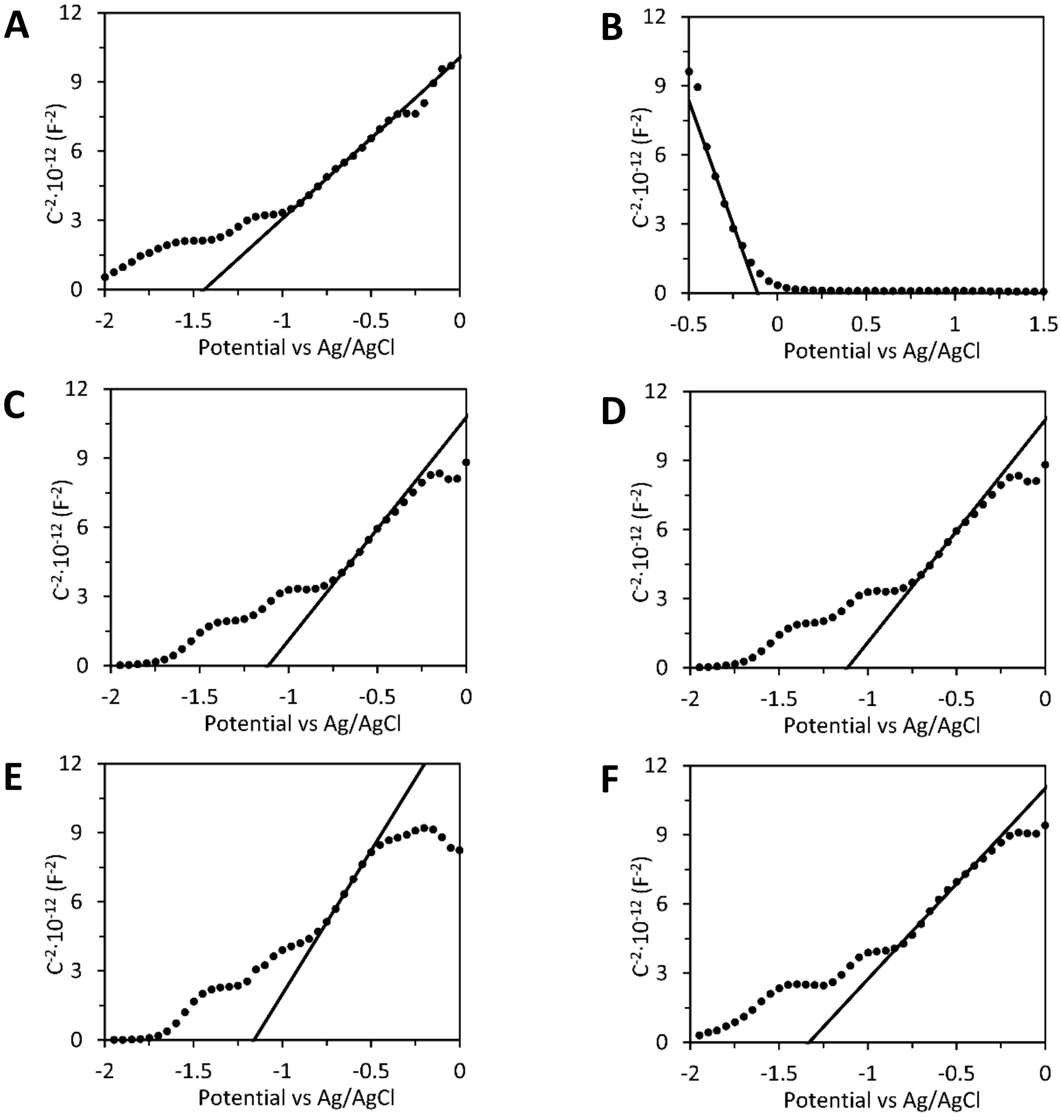

Fig. S8: Mott-Schottky plots of prepared samples obtained from impedance measurement of **(A)** gCN, **(B)** F@FC, **(C)** F@FC-gCN (1:5), **(D)** F@FC-gCN (1:10), **(E)** F@FC-gCN (1:15), and **(F)** F@FC-gCN (1:20) composite.

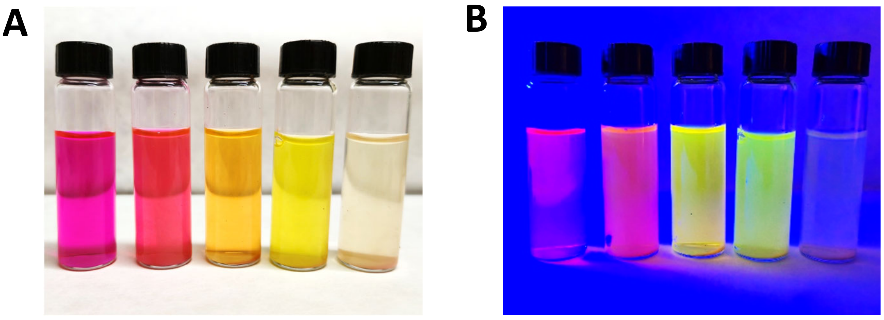

Fig. S9: Color of Rhodamine B solution within the photodegradation process for 0, 15, 30, 45 and 60 minutes **(A)** picture taken under normal lightning and **(B)** under UV light. Depiction of formatting yellowfluorescent intermediate product under photocatalytic degradation route.

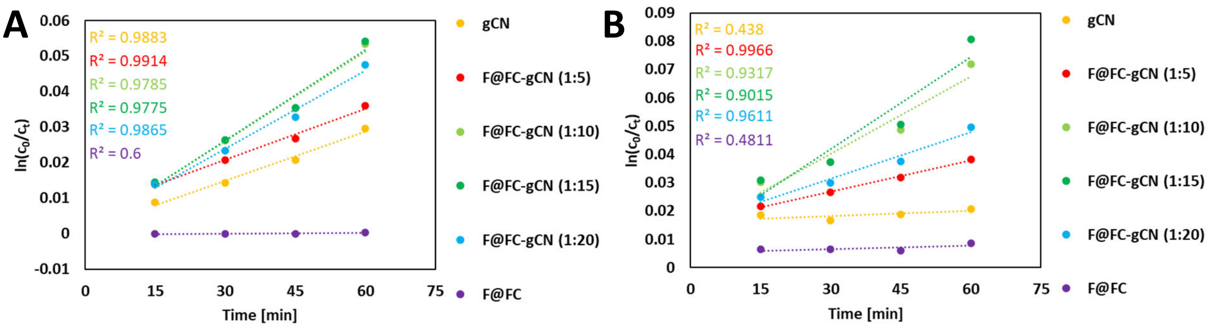

Fig. S10: The reaction rate constant plots with R2 coefficients of determination for **(A)** photocatalytic degradation, and **(B)** Photo-Fenton degradation.

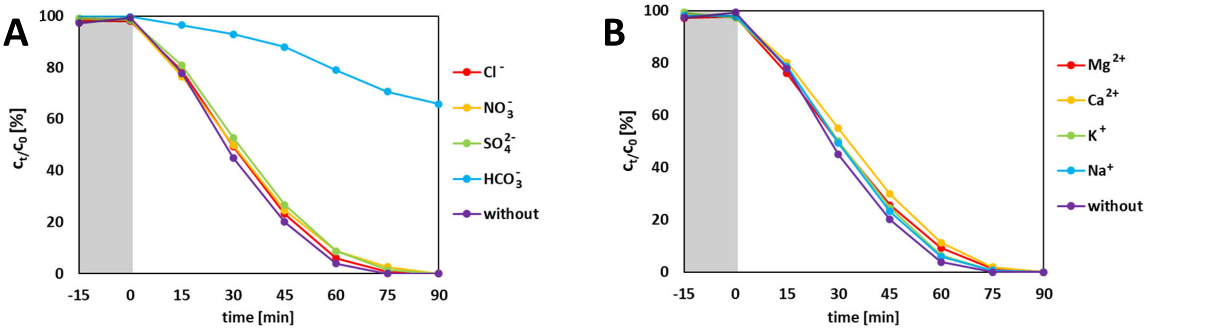

Fig. S11: **(A)** The effect of inorganic anions on the photocatalytic degradation of RhB using F@FC-gCN (1:15); and **(B)** the effect of inorganic cations.

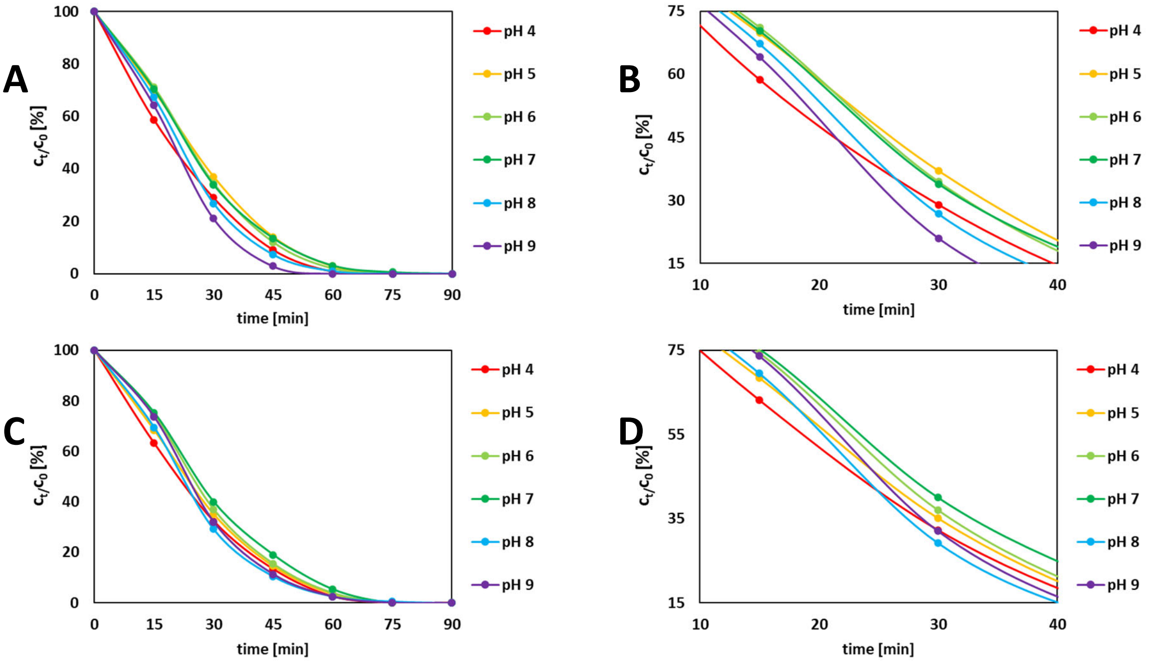

Fig. S12: The effect of pH on the Photo-Fenton degradation of Rhodamine B using the F@FC-gCN (1:15) composite at two hydrogen peroxide concentrations: **(A)** 0.05M H_2O_2 and **(C)** 0.01M H_2O_2. Images **(B)** and **(D)** represent magnified views of the 10-40 min reaction period for the respective experiments to enhance distinction of the degradation kinetics.

Table S1: Positions of peaks in XPS spectrum for samples composite F@FC-gCN 1:15, pure F@FC, and pure gCN.

Element	Peak	Binding energy [eV]			Shift in binding energy [eV]	
		F@FC-gCN 1:15	F@FC	gCN	From F@FC to composite	From gCN to composite
Fe	Fe 2p3 (III)	710.41	710.05		+0.36	
Ce	Ce 3p9 (IV)	882.45	881.68		+0.77	
	Ce 3p5 (III)	898.71	897.54		+1.17	
O	C-O	532.85	533.17	532.31	-0.32	+0.54
	C=O	531.44	530.64		+0.80	
	Metal oxide	529.37	528.69		+0.68	
C	C-C	284.57		284.83		-0.26
	C-O	285.85		286.15		-0.30
	N=C-N	287.88		287.97		-0.09
	π-π	2893.43		293.52		-0.09
N	C=N-C	398.39		398.50		-0.11
	$N-C_3$	400.30		400.42		-0.12
	π-π	404.20		404.32		-0.12

Table S2: Summary of reaction rate constants for all experiments in times 15, 30, 45 and 60 minutes for photocatalytic degradation. In the standard procedure, 0.5 g/L of the F@FC-gCN (1:15) photocatalyst was used to degrade 20 mL of RhB solution at a concentration of 30 ppm.

Figure	Change of conditions	Reaction rate constant in time of			
		15 min	30 min	45 min	60 min
Influence of CFO and gCN ratio in the resulted composite (Fig. 7A)	gCN	0.0088	0.0143	0.0208	0.0296
	F@FC-gCN (1:5)	0.0140	0.0208	0.0268	0.0360
	F@FC-gCN (1:10)	0.0144	0.0262	0.0352	0.0534
	F@FC-gCN (1:15)	0.0145	0.0264	0.0355	0.0541
	F@FC-gCN (1:20)	0.0140	0.0234	0.0329	0.0476
	F@FC	0.0000	0.0000	0.0000	0.0004
Influence of initial concentration of photocatalyst (Fig. 7B)	0.10 g/l	0.0061	0.0083	0.0098	0.0119
	0.25 g/l	0.0079	0.0127	0.0167	0.0213
	0.50 g/l	0.0145	0.0264	0.0355	0.0541
	1.00 g/l	0.0342	0.0568	0.1057	0.0886
	1.50 g/l	0.0484	0.0860	0.1197	-
Influence of concentration of initial RhB (Fig. 7C)	10 ppm	0.0371	0.0696	0.0881	-
	20 ppm	0.0278	0.0445	0.0817	-
	30 ppm	0.0145	0.0264	0.0355	0.0541
	40 ppm	0.0132	0.0193	0.0279	0.0388
Influence of pH (Fig. 7D)	pH 4	0.0176	0.0254	0.0353	0.0539
	pH 5	0.0110	0.0172	0.0248	0.0352
	pH 6	0.0091	0.0145	0.0208	0.0287
	pH 7	0.0058	0.0083	0.0115	0.0152
	pH 8	0.0086	0.0102	0.0125	0.0147
	pH 9	0.0046	0.0067	0.0091	0.0110
Influence of quenching of $\bullet O_2^-$, $\bullet OH$, h^+, and e^- (Fig. 7E)	L-histidine	0.0050	0.0074	0.0084	0.0087
	IPA	0.0162	0.0246	0.0383	0.0582
	EDTA-2Na	0.0140	0.0108	0.0104	0.0103
	K_2CrO_4	0.0115	0.0147	0.0170	0.0209
	without	0.0145	0.0264	0.0355	0.0541
Influence of anions presence (Fig. S9A)	Cl^-	0.0146	0.0228	0.0320	0.0466
	NO_3^-	0.0168	0.0227	0.0307	0.0403
	SO_4^{2-}	0.0132	0.0208	0.0289	0.0403
	HCO_3^-	0.0021	0.0023	0.0027	0.0039
	without	0.0145	0.0264	0.0355	0.0541
Influence of cations presence (Fig. S9B)	Mg^{2+}	0.0168	0.0226	0.0297	0.0394
	Ca^{2+}	0.0133	0.0192	0.0264	0.0360
	K^+	0.0143	0.0221	0.0305	0.0453
	Na^+	0.0146	0.0228	0.0320	0.0466
	without	0.0145	0.0264	0.0355	0.0541

Table S3: Summary of reaction rate constants for all experiments in times 15, 30, 45 and 60 minutes for Photo-Fenton degradation. In the standard procedure, 0.5 g/L of the F@FC-gCN (1:15) photocatalyst was used to degrade 20 mL of RhB solution at a concentration of 30 ppm and H_2O_2 concentration of 0.05 M.

Figure	Sample	Reaction rate constant in time X [min^{-1}]			
		15 min	30 min	45 min	60 min
Influence of CFO and gCN ratio in the resulted composite (Fig. 8A)	gCN	0.0186	0.0166	0.0188	0.0208
	F@FC-gCN (1:5)	0.0217	0.0266	0.0319	0.0382
	F@FC-gCN (1:10)	0.0301	0.0373	0.0486	0.0720
	F@FC-gCN (1:15)	0.0310	0.0374	0.0507	0.0807
	F@FC-gCN (1:20)	0.0250	0.0299	0.0375	0.0497
	F@FC	0.0064	0.0066	0.0061	0.0086
Influence of H_2O_2 concentration using CFO-gCN (1:15) composite (Fig. 8B)	0.0 M	0.0162	0.0246	0.0346	0.0534
	0.05 M	0.0310	0.0373	0.0507	0.0807
	0.1 M	0.0404	0.0521	0.0801	0.0835
	0.5 M	0.0669	0.0930	-	-
	0.5 M - dark	0.0024	0.0008	0.0007	0.0124
Influence of H_2O_2 concentration using pure gCN (Fig. 8C)	0.0 M	0.0000	0.0002	-0.0011	-0.0001
	0.05 M	0.0064	0.0066	0.0061	0.0086
	0.1 M	0.0064	0.0092	0.0106	0.0126
	0.5 M	0.0119	0.0196	0.0288	0.0372
	0.5 M - dark	0.0028	0.0023	0.0022	0.0030
Influence of H_2O_2 concentration using pure CFO (Fig. 8D)	0.0 M	0.0088	0.0143	0.0200	0.0289
	0.05 M	0.0186	0.0166	0.0188	0.0208
	0.1 M	0.0194	0.0250	0.0286	0.0447
	0.5 M	0.0274	0.0365	0.0573	0.0874
	0.5 M - dark	0.0047	0.0012	0.0008	0.0123
Influence of H_2O_2 concentration on RhB photolysis (Fig. 8E)	0 M	-0.0030	-0.0005	0.0005	0.0011
	0.5 M	0.0114	0.0123	0.0147	0.0172
	0.5 M - dark	0.0005	0.0009	0.0003	0.0001
Influence of pH (Fig. 8F)	pH 4	0.0355	0.0413	0.0532	0.0796
	pH 5	0.0239	0.0331	0.0436	0.0591
	pH 6	0.0226	0.0355	0.0472	0.0659
	pH 7	0.0234	0.0360	0.0444	0.0580
	pH 8	0.0264	0.0439	0.0583	0.0774
	pH 9	0.0296	0.0520	0.0790	-
Influence of quenching of $•O_2^-$, •OH, and h^+ with H_2O_2 concentration of 0.1 M (Fig. 8G)	L-histidine	0.0063	0.0082	0.0104	0.0104
	IPA	0.0233	0.0332	0.0474	0.0822
	EDTA-2Na	0.0244	0.0224	0.0244	0.0269
	without	0.0404	0.0521	0.0801	0.0835

Table S4: The comparative table of photodegradation activity (PDA) of recently reported graphitic carbon nitride based photocatalysts.

Material	Degradation conditions	Sample	PDA t_{30} [%]	Cit.
**CeO_2/g-C_3N_4**	c_{cat}: 0.2 g/L<br>c_{pol}: 10 ppm<br>natural solar radiation<br>(90 – 110 K)<br>Rhodamine B	g-C_3N_4	17.0	[1]
		CeO_2	5.8	
		CNCe15	60.3	
**CeO_2/g-C_3N_4**	c_{cat}: 0.04 g/L<br>c_{pol}: 10 ppm<br>200W Xe lamp<br>Rhodamine B	g-C_3N_4	16.0	[2]
		CeO_2	14.1	
		CG2	24.8	
**Fe_2O_3/g-C_3N_4**	c_{cat}: 0.25 g/L<br>c_{pol}: 20 ppm<br>300W Xe lamp<br>Rhodamine B	g-C_3N_4	23.7	[3]
		Fe_2O_3	2.3	
		g-C_3N_4/Fe_2O_3-2	50.8	
**Fe_3O_4/CeO_2 nanohybrids over 2D g-C_3N_4**	c_{cat}: 0.24 g/L<br>c_{pol}: 10 ppm<br>11,780 lx LED lamp<br>Bismarck Brown R<br>**5 µl H_2O_2**	C1@GCN	28.0	[4]
		$C_1F_{0.2}$@GCN	73.2	
**Fe_2O_3/g-C_3N_4**	c_{cat}: 0.1 g/L<br>c_{pol}: 5 mg/L<br>65W CFL lamp<br>(125 W/m^2)<br>Rhodamine B	g-C_3N_4	41.3	[5]
		Fe_2O_3	51.7	
		Fe_2O_3/g-C_3N_4	66.5	
**Fe_2O_3@Fe-CeO_2 gCN**	c_{cat}: 0.5 g/L<br>c_{pol}: 30 mg/L<br>white LED light<br>(5920 lm, 6500 K)<br>Rhodamine B	g-C_3N_4	35	This study
		Fe_2O_3@Fe-CeO_2	0.6	
		F@FC-gCN (1:15)	55	
	– 〃 –<br>0.1 mol/l H_2O_2	g-C_3N_4	39.2	
		Fe_2O_3@Fe-CeO_2	18.1	
		F@FC-gCN (1:15)	67.4	

67. Alsulmi, A.; Mohammed, N.N.; Hassan, M.M.; Eltawil, M.A.; Amin, A.E.; Fahmy, M.; Sultan, A.; Ahmed, M.A. Rational Engineering of S-Scheme CeO2/g-C3N4 Heterojunctions for Effective Photocatalytic Destruction of Rhodamine B Dye under Natural Solar Radiations. *Colloids and Surfaces A: Physicochemical and Engineering Aspects* **2024**, *689*, 133683, doi:10.1016/j.colsurfa.2024.133683.

1. Shoran, S.; Chaudhary, S.; Sharma, A. Photocatalytic Dye Degradation and Antibacterial Activities of CeO2/g-C3N4 Nanomaterials for Environmental Applications. *Environmental Science and Pollution Research* **2022**, *30*, 98682–98700, doi:10.1007/s11356-022-23815-x.
2. Wang, J.; Zuo, X.; Cai, W.; Sun, J.; Ge, X.; Zhao, H. Facile Fabrication of Direct Solid-State Z-Scheme g-C_3 N_4 /Fe_2 O_3 Heterojunction: A Cost-Effective Photocatalyst with High Efficiency for the Degradation of Aqueous Organic Pollutants. *Dalton Transactions* **2018**, *47*, 15382–15390, doi:10.1039/C8DT02893A.
3. Gupta, S.V.; Ahmaruzzaman, Md. Designing of a Magnetically Recoverable ($Ce^{+3, +4}$ /Fe^{+2} . Fe^{+3})$_{oxide}$ @ 2D g-C_3 N_4 Surface for Enhanced Photo-Fenton Catalytic Degradation of Bismarck-Brown R and Congo Red Dyes: Kinetics, Optical Properties,and Mechanistic Pathway Studies. *International Journal of Environmental Analytical Chemistry* **2024**, *104*, 6997–7028, doi:10.1080/03067319.2022.2159819.
4. Singh, J.; Basu, S. Synthesis of Mesoporous Magnetic Fe2O3/g-C3N4 Monoliths for Rhodamine B Removal. *Microporous and Mesoporous Materials* **2020**, *303*, 110299, doi:10.1016/j.micromeso.2020.110299.
